# Stochastic modeling indicates that aging and somatic evolution in the hematopoietic system are driven by non-cell-autonomous processes

**DOI:** 10.18632/aging.100707

**Published:** 2014-12-17

**Authors:** Andrii I. Rozhok, Jennifer L. Salstrom, James DeGregori

**Affiliations:** ^1^ Department of Biochemistry and Molecular Genetics, University of Colorado School of Medicine, Aurora, CO 80045; ^2^ Integrated Department of Immunology, University of Colorado School of Medicine, Aurora, CO 80045; ^3^ Department of Pediatrics, University of Colorado School of Medicine, Aurora, CO 80045; ^4^ Department of Medicine, Section of Hematology, University of Colorado School of Medicine, Aurora, CO 80045

**Keywords:** hematopoiesis, carcinogenesis, leukemogenesis somatic evolution, fitness, driver mutation, microenvironment, adaptation

## Abstract

Age-dependent tissue decline and increased cancer incidence are widely accepted to be rate-limited by the accumulation of somatic mutations over time. Current models of carcinogenesis are dominated by the assumption that oncogenic mutations have defined advantageous fitness effects on recipient stem and progenitor cells, promoting and rate-limiting somatic evolution. However, this assumption is markedly discrepant with evolutionary theory, whereby fitness is a dynamic property of a phenotype imposed upon and widely modulated by environment. We computationally modeled dynamic microenvironment-dependent fitness alterations in hematopoietic stem cells (HSC) within the Sprengel-Liebig system known to govern evolution at the population level. Our model for the first time integrates real data on age-dependent dynamics of HSC division rates, pool size, and accumulation of genetic changes and demonstrates that somatic evolution is not rate-limited by the occurrence of mutations, but instead results from aged microenvironment-driven alterations in the selective/fitness value of previously accumulated genetic changes. Our results are also consistent with evolutionary models of aging and thus oppose both somatic mutation-centric paradigms of carcinogenesis and tissue functional decline. In total, we demonstrate that aging directly promotes HSC fitness decline and somatic evolution via non-cell-autonomous mechanisms.

## INTRODUCTION

Aging and cancer are widely considered to be rate-limited by the accumulation of phenotype-altering mutations and the incidence of oncogenic driver mutations, respectively. The Somatic Mutation Theory of Aging postulates that the accumulation of somatic DNA alterations with age largely accounts for aging phenotypes [1-4]. The accumulation of somatic mutations and epigenetic changes has also been proposed to be a major cause of age-related stem cell decline, such as for hematopoietic stem cells (HSC) [5, 6]. Similarly, the modern Multi-Stage Model of Carcinogenesis argues that cancer incidence is driven by the occurrence of successive cancer driver mutations in individual clones, leading to a step-wise acquisition of progressively more malignant phenotypes [7-10]. Each driver mutation is thought to confer a certain fitness advantage over the rest of the stem cell pool, leading to expansion of that cell's progeny. This clonal expansion then increases the chance that the next driver mutation will happen in a cell containing the initial oncogenic mutation, thereby promoting progression to a multi-driver malignant cell phenotype.

Current models of cancer operate with the assumption that the extent of fitness effects is a defined property of oncogenic mutations [11-13]. From this perspective, oncogenic mutations are capable of driving somatic evolution upon their occurrence, and their occurrence thus determines the timing of a multi-stage process of selection for pre-malignant clones, eventually leading to cancer. However, this assumption is markedly discrepant with evolutionary theory, whereby fitness is a dynamic property of phenotype and is defined and extensively modified by environment [14, 15]. From the perspective of modern evolutionary theory, genetic changes may have a defined effect on phenotype, but the resulting changes in fitness are not defined and are environment-dependent.

The current paradigm of cancer rests on early assumptions that mutation accumulation over lifetime is linear [16]. However, more recent evidence from humans and other mammals indicates that roughly half of all mutations and epigenetic changes (including potentially oncogenic events) in HSC and other tissues accumulate early in life before full body maturation [17-19], consistent with a concomitant rapid slowdown in stem cell division rates post-development [20, 21]. Indeed, oncogenic driver mutations are frequently detected in healthy tissues of individuals of different ages without a diagnosis of cancer, suggesting that there may be a significant delay between the occurrence of oncogenic drivers and the actual onset of somatic evolutionary processes driven by these mutations [22-24]. This early-life accumulation of a substantial portion of genetic damage in tissues is also at odds with the delay in body fitness decline and aging until post-reproductive periods [25-27].

Thus, both cancer incidence and aging are delayed until the post-reproductive period of lifespans, being significantly offset from the timing when a substantial portion of phenotype-altering genetic damage occurs. Such a delay is explained by evolutionary models of aging via the reduced investment in tissue maintenance, as selection at the population/germline level becomes progressively relaxed with progression into post-reproductive ages [28, 29]. The tremendous diversity of lifespans within physiologically similar groups, such as mammals, contradicts the mutation-centric views and instead suggests that aging is an evolved process regulated above the cell-intrinsic level. Indeed, the role of DNA damage as a cause of aging has been challenged based on evidence supporting the idea that aging is a systemic process [30]. Moreover, the exponentially increasing incidence of cancers in the latter half of potential lifespans is nearly universal across mammalian taxa with different lifespans [31]. Even within the same species, a roughly similar age-dependent incidence pattern is typical of diverse cancers originating from cell pools of drastically different sizes and cell division dynamics. An increased incidence in late-life is also typical across cancers requiring different numbers of driver mutations, including those thought to require only a single oncogenic mutation, such as chronic myeloid leukemia (CML) in chronic phase [32]. Finally, the higher incidence of some cancers in the first few years of human life relative to older juvenile and young adult years is difficult to explain from the simple perspective of time-dependent mutation accumulation. Thus, cancer incidence patterns across lifespans cannot be explained by mutation accumulation alone, regardless of the type of cancer or organism studied.

Importantly, currently accepted models of age-dependent carcinogenesis have not taken into account a number of key age-altered factors that should interact in a complex manner to shape somatic evolution, such as the dynamics of mutation accumulation, changing size of stem cell pools with body growth, alterations in stem cell division rates, and the role of tissue microenvironments [12, 13, 16, 33-35]. Indeed, these previous studies model cancer evolution as a purely cell intrinsic process. Given that species evolution is largely driven by environmental change, and clearly influenced by population size, reproduction rates, and mutational variation, understanding the somatic evolution of cancers will require consideration of analogous factors.

An alternative model proposes that cancer development is rate-limited by the dynamics of age-dependent tissue fitness decline (and is thus tightly linked to the evolution of lifespan and aging), whereby cells in aged tissues of lower general fitness provide more room for positive selection for oncogenic events that have accumulated in tissues over a lifetime [23, 24, 34]. This model thus proposes that the fitness value of oncogenic events is limited and mostly negative in young highly fit tissues optimized by evolution at the germline level, but their fitness effects may increase in aged tissues, facilitating somatic evolution. Indeed, the fitness effects of many cancer drivers have been shown experimentally to vary in a microenvironment-dependent manner [35-40]. The importance of tissue microenvironment in regulating somatic evolution in animal tissues is supported by numerous modeling and experimental studies [22, 41-46]. Maintenance of tissue microenvironment could therefore be a powerful mechanism suppressing cellular fitness decline and somatic evolution through reproductive ages by impairing the competitive potential of cells with altered genotypes/phenotypes and thus increasing the likelihood of their elimination from a cell population [24, 34].

Here we explore the theoretical limits of the effects of mutations on cellular fitness decline and somatic evolution in HSC pools across human lifespans. The hematopoietic system is well established as a model for clonal dynamics [47-49]. We simulated these clonal dynamics using a Monte Carlo approach whereby the fate of each cell in the modeled HSC pool evolves independently based on defined initial parameters and the probability distributions that describe the change in these parameters, such as cell division frequency, cell fitness change, cell fate decisions, and mutation accumulation. The stochastic approach allowed us to model non-linear cell dynamics and the complex relationship between cell-autonomous and non-cell-autonomous processes that control somatic evolution and fitness dynamics within the HSC pool. Our results indicate that somatic evolution is suppressed early in life by stabilizing selection, which minimizes the positive fitness value of mutations. We demonstrate that the evolutionary concept of dynamic fitness values for genetic changes which is extensively modulated and defined by tissue microenvironment is necessary to explain the age-related rates of somatic evolution. Thus, somatic evolution in HSC pools and consequently hematopoietic malignancies are not rate-limited by the occurrence of oncogenic mutations and are instead driven by altered positive selection in aging tissues for previously generated genetic and phenotypic cell diversity.

## RESULTS

### Conceptual basis of the model

We assume that all possible random somatically heritable genetic and epigenetic changes, collectively referred to hereafter as mutations, have a theoretical distribution of fitness effects (DFE) per cell division. Neither the shape nor the variance of this DFE is known for HSCs. At the organismal population level, most mutations have either no or negligible phenotypic effects or are lethal (reviewed in [50]). Mutations that reduce fitness are much more frequent than those that confer a selective advantage. However, as somatic cells are subject to selection at both the animal population and tissue levels, the net DFE of somatic mutations that affects inter-cellular competition and somatic evolution at the tissue level could differ from DFEs typically observed at the animal population level.

**Figure 1:**
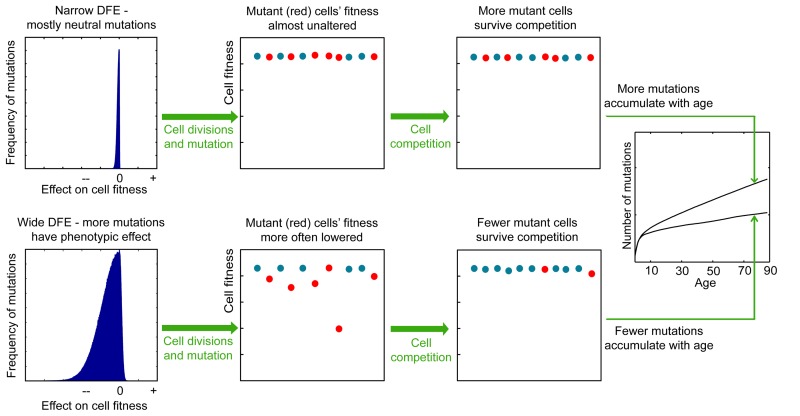
The proposed effect of the mutation DFE on the slope of mutation accumulation in stem cell pools with age *Lower panels*, a wide DFE leads to a large fitness differential among cells in the pool. Mutations affecting phenotype are known to have mostly negative fitness effects accounting for the large negative tail in the wide DFE. Cells harboring negative mutations will be eliminated by cell competition effects. These cells are likely to be cells that have undergone a greater number of divisions and thereby possess more mutations. Consequently, mutations accumulate more slowly in the population. *Upper panels*: less frequent non-neutral mutations (a narrow DFE) generate less fitness differential in the pool, and thus the frequency of survival of mutated cells is dominated by drift (chance) rather than selection; the average number of mutations per cell will accumulate faster, at a rate that more closely reflects mutation occurrence and thus cell division frequency.

To infer the nature of DFE in HSC pools, we applied the logic that mutation fitness effects will affect competition and selection processes by creating a fitness differential in a population of cells. A narrow DFE with mostly neutral mutations will result in a largely drift-driven population, where the chance of survival is random (Fig. 1; upper panels). A wide DFE, where a larger proportion of mutations have fitness effects, will engender a broader fitness differential among cells within a population. This fitness differential lends itself to natural selection, wherein cells with disadvantageous phenotypes are less likely to survive into subsequent generations. As most functional mutations are likely to decrease fitness (residing in the negative tail of the DFE), we reasoned that variance of DFE should have an effect on the slope of mutation accumulation in HSCs, whereby wide variance (more functional mutations) should act to purge mutated cells with decreased fitness from the pool and thus act to lower the speed of mutation accumulation in the population (Fig. 1; lower panels).

Mutation accumulation in the Tier 3 genome can be used as a marker for overall mutation accumulation rates. Tier 3 represents (to best approximations) the non-conserved, non-coding, and non-repetitive sequences of the genome, spanning ~1 billion base pairs [51]. The vast majority of Tier 3 DNA should therefore not be under selection (particularly in somatic cells), and mutations in Tier 3 should (with rare exceptions) not alter cellular phenotype. But the accumulation of these passenger mutations will depend on the intensity of purifying selection acting on functional mutations (including epigenetic alterations) as shown in Fig. 1, and can be used to infer the intensity of selection and thus the parameters of mutation DFE acting on HSC. Basically, increased accumulation of mutations with detrimental phenotypic effects will confer a fitness cost to cell division. We will use our model to determine the parameters of mutation DFE and rate increase over a human lifetime that can replicate experimentally determined mutation accumulation in the Tier 3 genome of HSC.

Since the most common leukemias appear to initiate in HSC [52-56], the initial clonal expansions of an oncogenically-initiated HSC should be rate-limiting for the occurrence of successive oncogenic events in the clonal context and eventually for the development of hematopoietic malignancies. We will thus also ask if the estimated realistic parameters for mutation DFE are concomitantly permissive for age-dependent exponential increases in the extent of clonal expansions in the HSC pool. The rates of somatic evolution (extent of mutation-driven clonal expansions) is a major factor defining the ultimate odds and frequency of leukemia over time/age, as can be inferred from the following equation integrating the probability that a set of *n* successive oncogenic drivers {d1, …, dn} will happen in any single cell in a stem cell pool over a lifetime (Eq. 1):

$$P_{d1\ldots dn}\left(t\right)=\text{D}\left(t\right)\times \int_{0}^{t}\left(\prod_{i=1}^{n}p_{i}\right)\left(t\right)dt$$ (1)

where *P*_d1_…_dn_(t) is the probability of acquiring *n* drivers in one cell by time *t*, D(t) is the total number of cell divisions that have happened in the pool by time *t*, and *p_i_* is the probability of acquiring a driver *i* ∈ {d_1_, …, d_n_} per cell per division as a linear function of the total number of mutations per cell per division.

The chances of acquiring a set of *n* drivers in any given cell in the pool over time depend on two factors: mutation rate and the total number of cell divisions by the time in question. From Eq. 1, it is evident that cell proliferation driven by acquisition of a fitness advantage will have a much more dramatic effect on the final probability of the whole set of drivers occurring within one genetic context compared to mutation rate. While a change in mutation rate can lead to a linear increase in this probability, the expansion of a selectively advantageous clone will elevate the probability of occurrence of subsequent driver mutations within a cell of this clone/genetic context exponentially. As Eq. 1 defines the probability density function of *n* drivers happening in any cell in the pool over time, clones making up a greater share of the pool will harbor proportionally more dividing cells and have proportionally higher chances of this set of drivers happening in a cell within the clone. Based on this logic, we assume that the shape of the age-dependent incidence of leukemia will mostly be determined by the age-dependent magnitude of selection-driven clonal expansions possible under given parameters for mutation DFE and rate. Therefore, we asked what mutation parameters are compatible with both the reported slope of mutation accumulation in the Tier 3 genome and with exponential increases in the magnitude of clonal expansions (increased positive selection) that replicate the shape of the age-dependent leukemia incidence curve.

### Architecture of the model

**Figure 2:**
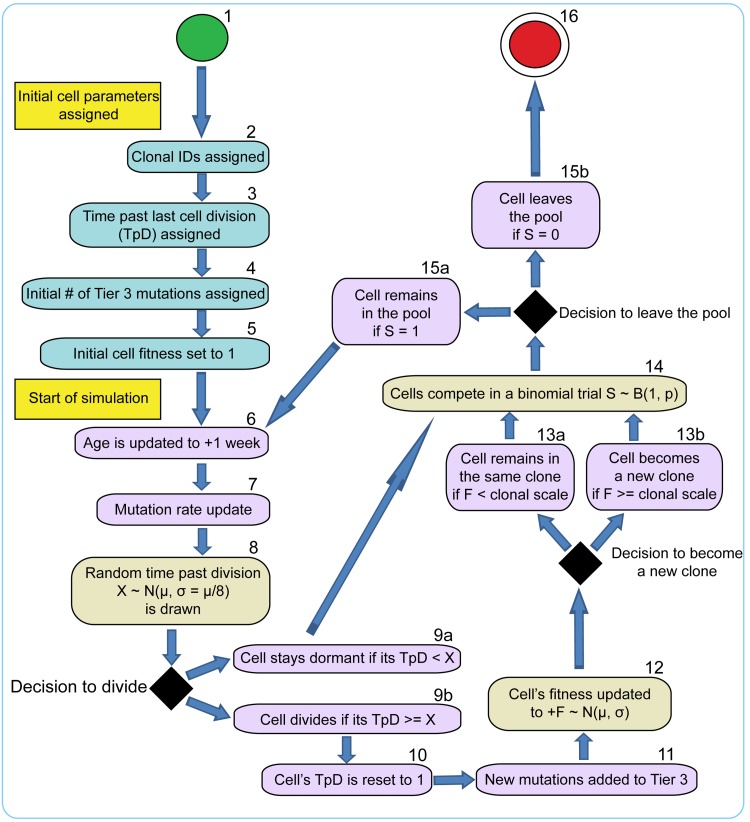
Stochastic model of HSC cell fate decisions 1 – initial cells (a total of 300); 2-5 – initial cell parameters; 6-15 – cell fate decisions during model cycle updated in “weekly” increments throughout the simulated human 85 year lifespan; 16 – cell leaves the pool. Timing of cells division (8), net fitness change per division (12), and fitness-based competition for niche space (14) are defined in stochastic trials based on distributions of average division timing, mutation DFE, and fitness-dependent stochastic competition scheme, respectively.

**Figure 3:**
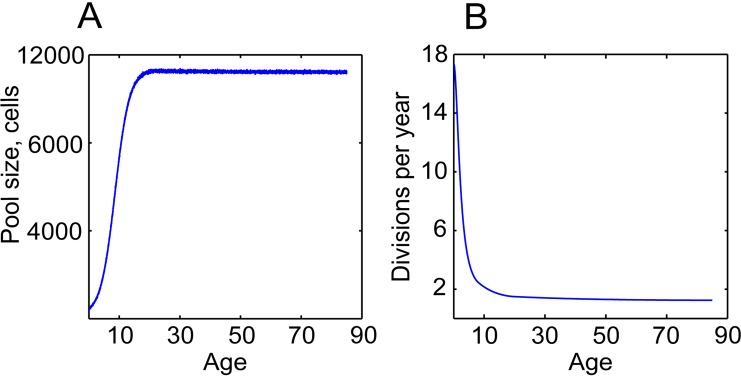
HSC division rates and pool size change dramatically throughout life (**A**) Dynamics of the pool size; the dynamics were inferred based on postnatal and adult HSC pool size estimates in [49, 72] (**B**) Average cell division frequencies; modeled based on estimates of HSC division rates at different ages in [20, 21, 49].

To fully investigate the many parameters governing somatic evolution in HSC pools, we designed a stochastic model to replicate HSC population dynamics, to simulate the impact of mutations in HSCs over human lifetimes, and to model the effects of tissue microenvironment on selection and clonal expansion within the HSC pool. This model is a stochastic, discrete time continuous parameter space model realized in a Monte Carlo experiment run in the Matlab programming environment (The MathWorks, Inc., Natick, Massachusetts). A chart of cell fate decisions in the simulated HSC pool during a model's run is shown in Fig. 2. The model starts with a matrix of the initial number of HSC, and each cell's state is updated on a “weekly” basis throughout the simulated lifespan of 85 years (4420 weeks). The weekly update included stochastic cell fate decisions to divide or stay dormant based on estimated HSCdivision rates throughout life (modeled based on published data; Fig. 3), and to stay in the pool or leave for whatever reason (such as death or differentiation) based on niche space availability, current number of competing cells at different ages (modeled based on published data for HSC pool size; Fig 3), and each cell's relative fitness. Cell fitness changed after each division stochastically, initially based only on mutation DFE. Cells that diverged in fitness more than a certain threshold from their parental cells upon division were designated as new clones, thus replicating functional (clonal) divergence of HSC in the pool with age (Fig. S1A-B). The code and detailed parameter description and justification are presented in Supplemental Methods.

### Fixed fitness effects of mutations cannot explain age-related functional decline and somatic evolution in HSC pools

**Figure 4:**
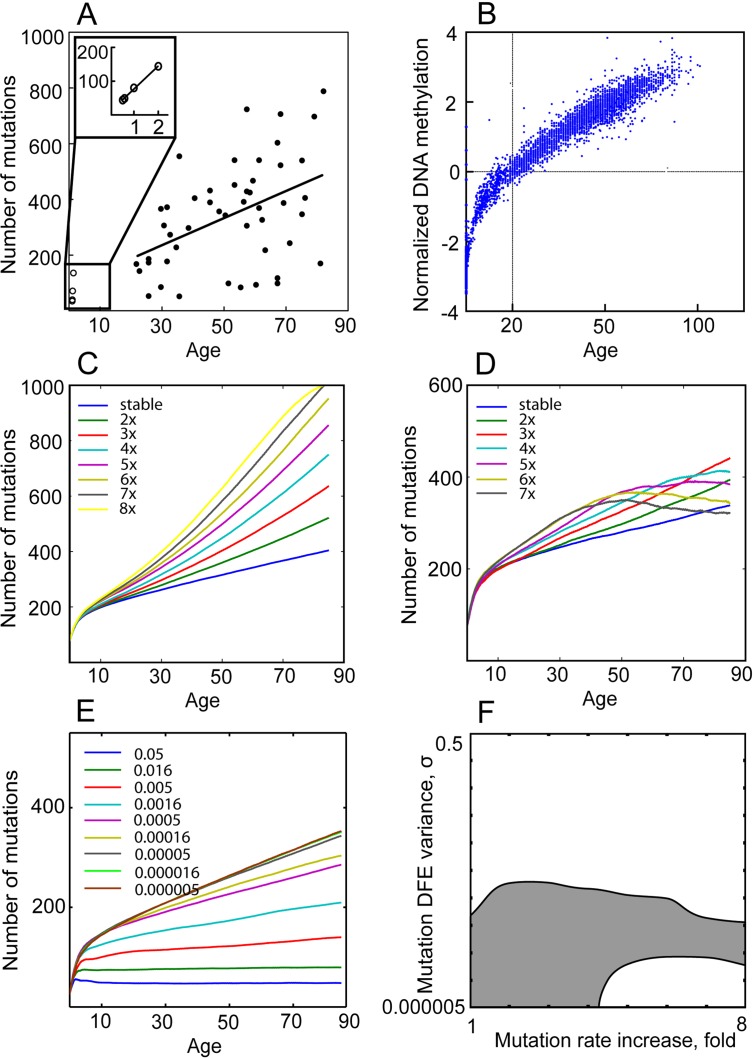
Mutation DFE affects the mutation accumulation slope (**A**) Mutation accumulation in the Tier 3 genome of AML (see inset for blowup of AMKL in children). (**B**) DNA methylation accumulation at neutral CpG islands of the human genome with age (from Horvath, 2013). (**C-D**) Simulated mutation accumulation in Tier 3 under mutation DFE variance σ= 0.000005 (C) and σ= 0.0005 (D) and different mutation rate fold increases over lifetime. (**E**) Simulated mutation accumulation in Tier 3 under stable mutation rate over lifetime and different mutation DFE variance. (**F)** The range (shaded) of mutation DFE variance (Y axis) and mutation fold rate increase over lifetime (X axis) that replicate WGS-derived slope of mutation accumulation in AML genomes and DNA methylation accumulation in the hematopoietic tissue within 95% confidence interval.

**Figure 5:**
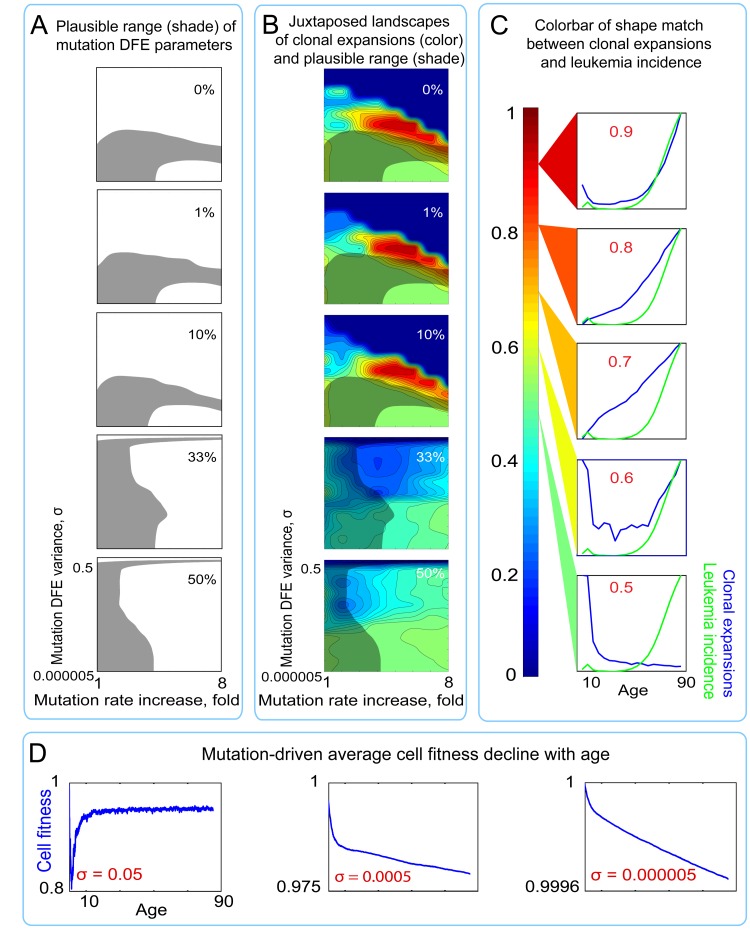
Landscapes of clonal expansions in the simulated pools under different parameters of mutation DFE when cell fitness is defined by mutations only (**A**) Shaded regions represent the plausible range of mutation DFE variance and rate increase over lifetime (for derivation see Fig. 4) under different proportions of mutations in the positive tail of the DFE. (**B**) Plots of shape match landscapes within the studied ranges of mutation DFE variance (σ; Y-axis) and mutation rate increase (X-axis). Colored landscapes represent age-dependent rates of somatic evolution as shown in the panel C. The proportion of mutation DFE variance in the positive tail is indicated in white text. Plausible ranges of mutation parameters from panel A are compared to mutation parameters that replicate exponentially increasing rates of somatic evolution that shape-match the leukemia incidence curve. (**C**) Color scale for panel B; colors represent the goodness of shape match between age-dependent leukemia incidence (green line) and simulated clonal expansions (blue line depicts the share of the most successful clone at any given time). (**D**) Age-dependent average cell fitness decline in the simulated pool for the indicated values of mutation DFE (σ).

We found mutation DFE variance (in standard deviations, denoted as σ) to influence the slope of mutation accumulation within the range σ= 5×10^−1^–5×10^−6^ and mutation rate increase from stable to up to 8-fold over lifetime (Fig. 4). As described above and outlined in Fig. 1, higher σwill suppress mutation accumulation, including in the Tier 3 genome, by essentially imposing a penalty on cell division. An increasing mutation rate effectively widens DFE variance per cell division following the rule of distribution sums [57] (Fig. S1C-D). We used these ranges for mutation rate increase and DFE variance over lifetime as the X and Y axes of a plane, with the Z axis represented by the model output of interest (more details on generating the surface plots can be found in Supplemental Methods). In this manner, we built surfaces of model outputs of interest (such as slope of mutation accumulation or magnitude of clonal expansions over lifetime) within the effective range of mutation DFE variance and mutation rate increase. Mutation DFE was always centered on zero, with the majority of mutations being neutral. Within this principal design of measuring the simulated model's output, we also tested a range of mutation DFE shapes (DFE tail ratios), with 0, 1, 10, 33, and 50% of mutations in the positive tail. A larger positive tail means a greater frequency of beneficial mutations. Note that even when 0% of mutation DFE is in the positive tail, fitness differential will still build up within the population (with some cells “less negatively” affected). We assessed three basic model outputs: 1) the Tier 3 mutation accumulation slope; 2) the maximum extent of somatic evolution possible at any given age measured as the percentage of the pool occupied by the most successful clone; and 3) the dynamics of average cell fitness in the pool, to see if the model replicates the general stem cell functional decline over lifetime. The reference Tier 3 mutation accumulation slope with age for AML (0.09162; 95% confidence interval, CI: 0.03759-0.1457; Fig. 4A; The Cancer Genome Atlas Research Network, 2013) was used to delineate the range of mutation DFE and rate parameters that reproduced the slope of DNA mutation accumulation in the Tier 3 genome of HSC. As argued by others [58], mutation accumulation in AML is thought to reflect mutation accumulation in individual HSC (see Supplemental Methods). We will hereafter call this range the *plausible range*, depicted as shaded areas in Fig. 4F and 5A,B, to signify the ranges of mutation DFE variance and rate parameters that are most likely to enclose real parameters. Notably, the slope of DNA methylation accumulation in a set of CpG sites in blood cells ([19]; 95% CI: 0.061-0.062; Fig. 4B) is well within the CI for Tier 3 mutations (Fig. S2), indicating that mutation accumulation rates for genetic and epigenetic changes may be similar (both being consistent with cell division kinetics; Fig. 3A). We registered maximum rates of somatic evolution throughout the lifespan to see what parameters of the mutation process are permissive for age-dependent exponential increases in the magnitude of mutation-driven clonal expansions that would resemble the age-dependent increase in leukemia incidence. As evident from Eq. 1, the frequency of leukemia should closely follow the extent of somatic evolution, and somatic evolution rates, in turn, are dependent on the frequency of functional phenotype-altering mutations, i.e. on the parameters of the mutation rate and DFE. Typical age-dependent curves of clonal expansions generated by the model under different mutation DFE are shown in the shape-match plots with leukemia incidence in Fig. 5C. Lastly, the average fitness decline was monitored to test if the experimentally observed ~2-3-fold decline in HSC fitness over lifetime [5, 59, 60] was replicated in the model output.

Results shown in Fig. 5B (see also Table S1A, mutation-only conditions) reveal a substantial discrepancy between conditions required to replicate re-alistic mutation accumulation (shaded areas in Fig. 5A,B) and those that replicate clonal expansions (rates of somatic evolution) resembling the age-dependent shape of leukemia incidence. We used 0-1 normalization of leukemia incidence and the simulated clonal expansion curves to compare their shapes by the mean root square error (MRSE) method, with shape similarity between 0 (no similarity) and 1 (perfect match) (see color code in Fig. 5C).

**Figure 6:**
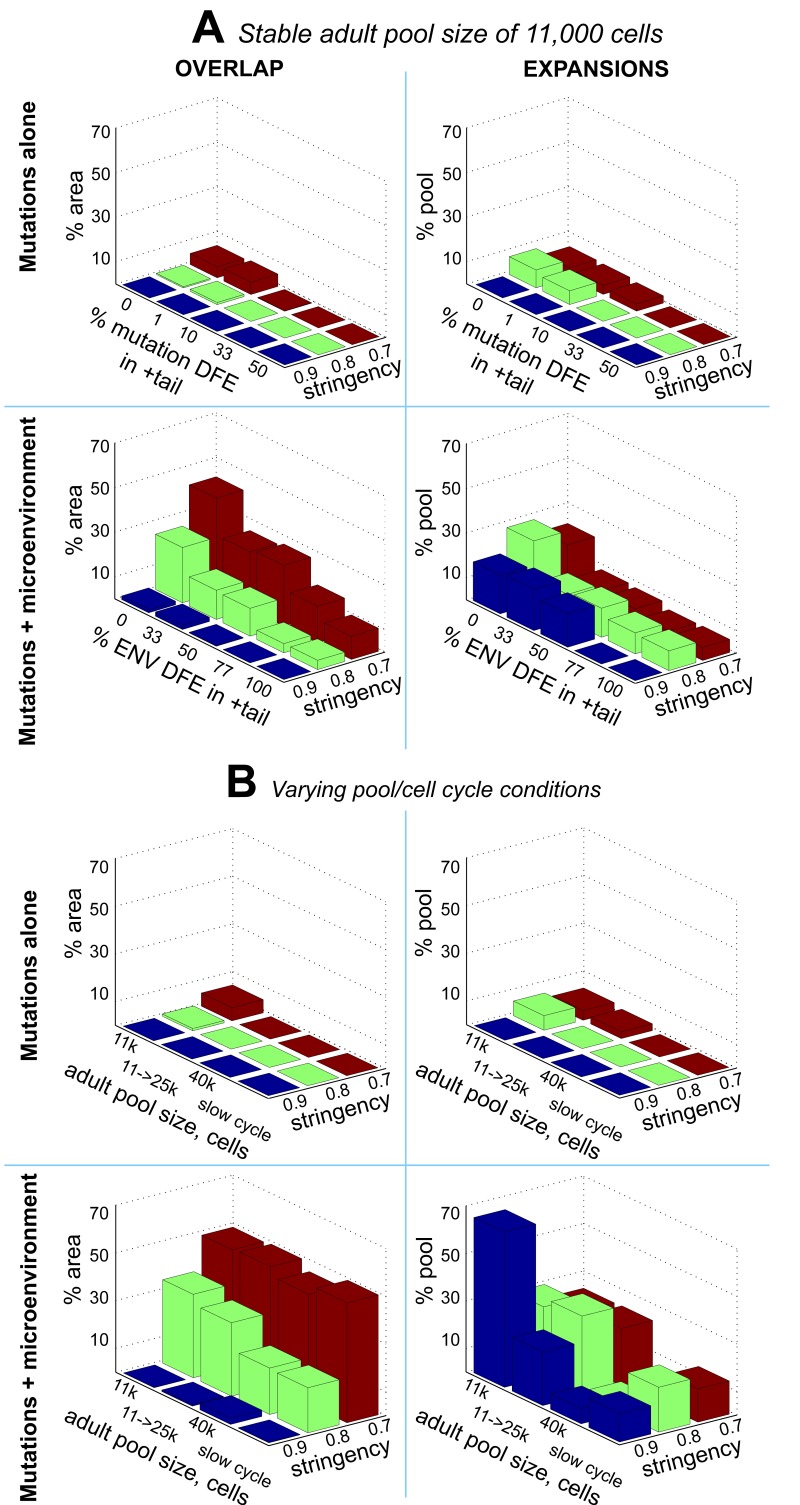
Clonal dynamics in the simulated HSC pools under different parameters of mutation and micro-environmental DFEs *OVERLAP* (left panels): % area of the *plausible range* of mutation parameters that allows age-dependent exponential clonal expansions under different *stringencies* (a minimum of 0.7, 0.8, or 0.9 shape match) of the expansions' match to the reference leukemia curve. *ENV DFE* – DFE imposed by microenvironment (explained in the text). *EXPANSIONS* (right panels): average magnitude of clonal expansions under different parameters of mutation and microenvironmental DFEs, measured as the % of pool occupied by the most successful clone at the end of the simulated life. (**A**) Comparison between mutation-alone (upper panels) and mutations + microenvironment (mutations + ENV) models (lower panels) under a stable adult HSC pool size of 11,000 cells; mutation DFE in the positive tail in all mutation + ENV conditions was set to 0%. (**B**) Same as **A** under different adult pool sizes and a slower cell cycling speed estimates (*slow cycle*); 11k->25k – the adult pool size increases over lifetime from 11,000 to 25,000 cells; mutation DFE in the positive tail in ALL conditions was set to 1% and ENV DFE in the positive tail was set to 0%. Numeric data is presented in Table S1.

A maximum of ~5% overlap was observed between the plausible range of mutation DFE and rate parameters replicating the empirically known slope of mutation accumulation (shaded areas in Fig. 5A,B) and the DFE range permissive for leukemia incidence-like shape of clonal expansions (red areas in Fig. 5B) under a liberal cutoff of ≥0.7 of shape similarities by MRSE. See Fig. 6 and Table S1A, “Mutations alone” conditions, for quantitative data. This overlap (the common set of mutation parameters needed to replicate both reference phenomena) only occurs in the middle range of mutation DFE variance (Y axis in plots in Fig. 5B) and requires a ≥4-fold mutation rate increase over lifetime (X axis in plots in Fig. 5B). As shown in Fig. 4D, under such combinations of mutation parameters, mutation accumulation is impeded or even reversed at later portions of life. The increasing mutation rate effectively increases DFE variance per cell division (see explanation Fig. S1A-B) and thus intensifies selection against mutant cells. Such a late-life slowdown in mutation accumulation or mutation purging has not been observed experimentally, and therefore we consider these combinations of mutation rate increase and DFE variance to be unrealistic.

Surprisingly, both the above-mentioned area of overlap and the magnitude of clonal expansions are drastically suppressed by an increased positive tail (more beneficial mutations) of the mutation DFE (Fig. 5B, Fig. 6, and Table S1A, “Mutations-alone” conditions). This suppression likely arises from the phenomenon known from bacterial populations as clonal interference, whereby an increasing number of high fitness clones mutually suppress the expansion of each other and that of other phenotypes [61, 62]. In our simulations, the strongest fitness gain (clonal expansions) over time based on sole action of mutations was possible under a zero to minute (1%) positive tail of the mutation DFE, consistent with the idea that stem cells reside at a local fitness peak (low probability of advantageous mutations), at least in young healthy individuals [34]. Therefore, the plausible range of mutation parameters is not permissive for significant exponential age-dependent increases in the rates of somatic evolution (clonal expansions).

The average cell fitness drop in the pool throughout lifespan also did not replicate the experimentally reported 2-3-fold decline in fitness for HSC in old age [5, 59, 60] and was restricted to a maximum of a few percent drop over lifetime throughout the whole range of conditions (Fig. 5D). Despite the mostly negative fitness effects of mutations, purifying selection appears to buffer the general cell fitness decline by purging mutation-affected cells from the pool. This effect of selection is particularly evident under wider mutation DFEs (Fig. 5D). Thus, accumulation of cell-autonomous damage is insufficient to account for HSC fitness decline in old age.

### Mutation fitness effects modified by microenvironment explain higher late-life rates of somatic evolution and fitness decline in HSC pools

Since we were unable to define a common set of mutation parameters that would recapitulate the experimentally observed mutation accumulation rates, somatic evolution/leukemia incidence, and HSC fitness decline with age, we tested an alternative, evolutionary model. For this purpose, we developed a model of bicomponent fitness effects exerted by the tissue microenvironment, containing a uniform and a randomly distributed part. The uniform component derives from the loss of tissue integrity with age, which should impact all cells in a tissue, as tissue microenvironment moves from its optimum towards a more degraded state. Roughly, this component dictates that cells generally have a lower fitness in a degraded (aged) microenvironment compared to the optimal (young) one. However, the degree of this influence should vary among cells based on the mutation-generated phenotypic diversity of cells. This variation is the distributed part of the microenvironmental effect that adds to selection processes by contributing to the fitness differential buildup in the cell population. We propose that this part of the environmental effect is distributed independently from the mutation DFE, so that any given phenotype that has a fitness advantage in an optimal microenvironment will not necessarily have this advantage in an altered environment (and vice versa), thus making the composite fitness effect of any given mutation context-dependent, just as it is in natural populations of organisms. Such a bicomponent effect of the tissue microenvironment is based on inference from the Sprengel-Liebig's system of limiting factors, initially known as Liebig's Law of the Minimum (summarized in [15]).

**Figure 7:**
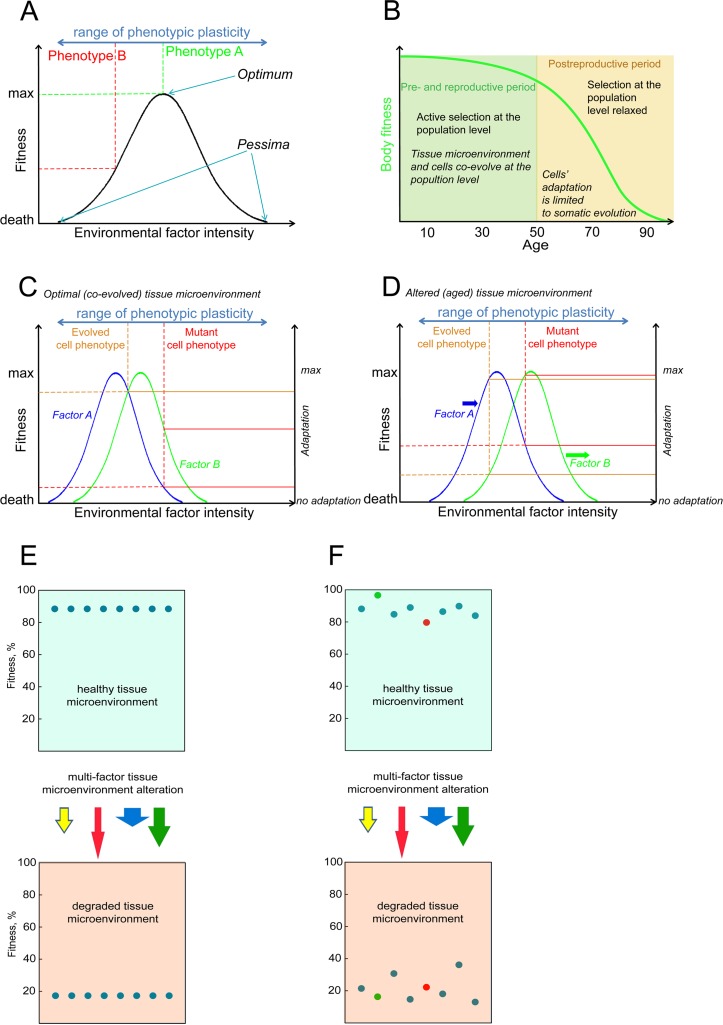
A schemata of a phenotype's fitness/survival determination in a complex environment **(A)** An illustration of the Shelford's Law of Tolerance: species survival decreases with a change in an environmental factor from the optimum towards its extrema within the species' tolerance range; selection drives adaptation of a species towards best survival optima (occupied by phenotype A in panel A). **(B)** Body fitness decline in humans and mammals is delayed until the post-reproductive period when selection for high fitness of the body is relaxed; at the animal population level microenvironment integrity is not supported by selection as age advances into the post-reproductive period and cells do not evolve to optimally perform in the altered microenvironment. **(C)** An example of cell fitness determination within a hypothetical bifactorial tissue microenvironment. The normal cell phenotype evolves for optimal performance at the animal population level, and thus the probability of somatic mutations that enhance cell performance is reduced. The evolved (“normal”) and mutant cell phenotypes have different degrees of adaptation to microenvironmental factors A and B (solid lines connecting to the right “adaptation” axis); following the Sprengel-Liebig Law of the Minimum, fitness of both phenotypes is limited by the factor each phenotype is least adapted to (dashed lines connecting to the left “fitness” axis). **(D)** An altered microenvironment of aging post-reproductive tissues (factor A and B intensities have changed). Selection at the animal population level is relaxed and neither of the cell phenotypes have evolved to an aged microenvironment (both are out of optima), but the fitness of the mutant phenotype may become higher in the altered microenvironment. **(E)** A phenotypically homogenous population of cells will decline in fitness in a degraded microenvironment, revealing the microenvironment's uniform component that affects fitness. **(F)** Phenotypic diversity creates fitness differential in the cell pool. In a degraded microenvironment relative fitness of cells may change and initially disadvantageous mutant phenotypes may gain in fitness relative to others (red cell in panel F) and vice versa (green cell), revealing the microenvironment's stochastic component affecting fitness independently of the initial fitness distribution.

The principle for how the Sprengel-Liebig's system defines the fitness of a given phenotype is shown in Fig. 7. In aggregate, any given phenotype has a certain degree of adaptation to each environmental factor so that the factor has its optimal intensity (e.g. optimal concentration) and extreme intensities, also called *pessima* (Fig. 7A). Fitness decreases as the intensity of a factor in the environment changes from the optimum to either of the *pessima* for a given phenotype, a phenomenon known as Shelford's Law of Tolerance [63]. Selection leads to improved adaptation so that populations consist of phenotypes for which a given environment is optimal. We argue that selection at the germline level should lead to co-evolution of stem cells and tissue microenvironment to optimize performance during pre- and reproductive periods (Fig. 7B). In a complex, multi-factorial environment a phenotype will have different degrees of adaptation to each particular factor, but the phenotype's net fitness will be limited by its adaptation to the factor it is least adapted to, called the limiting factor, following Sprengel-Liebig's Law of the Minimum (Fig. 7C) ([15, 64]). General adaptation to a complex environment leads to the evolution of phenotypes that have optimal net fitness (“evolved phenotype” in Fig. 7C), and this process reduces the likelihood that new phenotypes that arise from random mutations will improve fitness relative to the population as it becomes better adapted.

Environmental alterations lead to changes in factor intensities, which results in perturbation of the relative fitness of different phenotypes within a population and leads to selection for minority phenotypes that happen to have a better net fitness in an altered environment (Fig. 7D). Alteration of an environment reduces the average fitness of the population (Fig. 7E,F), revealing a uniform fitness effect on the population. However, in a phenotypically diverse population the exact degree of fitness change for any particular phenotype will depend on the phenotype's set of adaptations to particular environmental factors as shown in Fig. 7C,D, revealing a stochastic component of environmental effects which redistributes the relative fitness of particular phenotypes in a population independently from their initial distribution in an optimal environment. This general principle is the mechanism for how phenotypic effects of mutations are translated by environment into the phenotype's fitness.

To model this principle, we first generated a proposed curve of fitness decline, which describes a general non-linear ~3-fold fitness drop as a function of age using the following equation:

$$F\left(A\right)=F_{\max}-\left(\frac{F_{\max}-F_{\min}}{1+5200Ae^{-0.0031}}\right)$$ (2)

Where F is average HSC pool fitness, F_max_ is maximum initial fitness equal to 1, F_min_ is minimum end-life fitness equal to 0.3, and A is age in weeks. This curve was not intended to exactly replicate the natural fitness decline, as its shape is unknown, but reflects the general principle shown in Fig. 7B [65]. The overall 3-fold fitness reduction was chosen based on the 2-3 fold reductions in hematopoietic output per HSC observed for older humans and mice [5, 59, 60]. We used the first order derivative of this function to calculate the average fitness drop (Δ*F*) for any given discrete period. This amount was subtracted from each simulated cell in the pool at each weekly model update and represented the uniform part of the microenvironmental effects of cell fitness. To reproduce the distributed part of environmental effects, we then corrected the resulting fitness of each cell by an amount drawn from a normal distribution centered on zero and having variance σ (t) = Δ*F*(t)/2. As the chemical composition and physical conditions of tissue microenvironment are complex, their change with age will impact the existing mutation-generated functional diversity through an independent stochastic component, contributing to diversification of the relative fitness of different cells in a stochastic manner (Fig. 7F). In this way, mutations accumulated in a simulated cell lineage at any age are re-evaluated at each ”weekly” update of the model run; for example, mutations occurring during ontogeny could have an impact on HSC fitness later in life that differs greatly from their impact upon first occurrence (as the result of microenvironmental perturbations).

**Figure 8:**
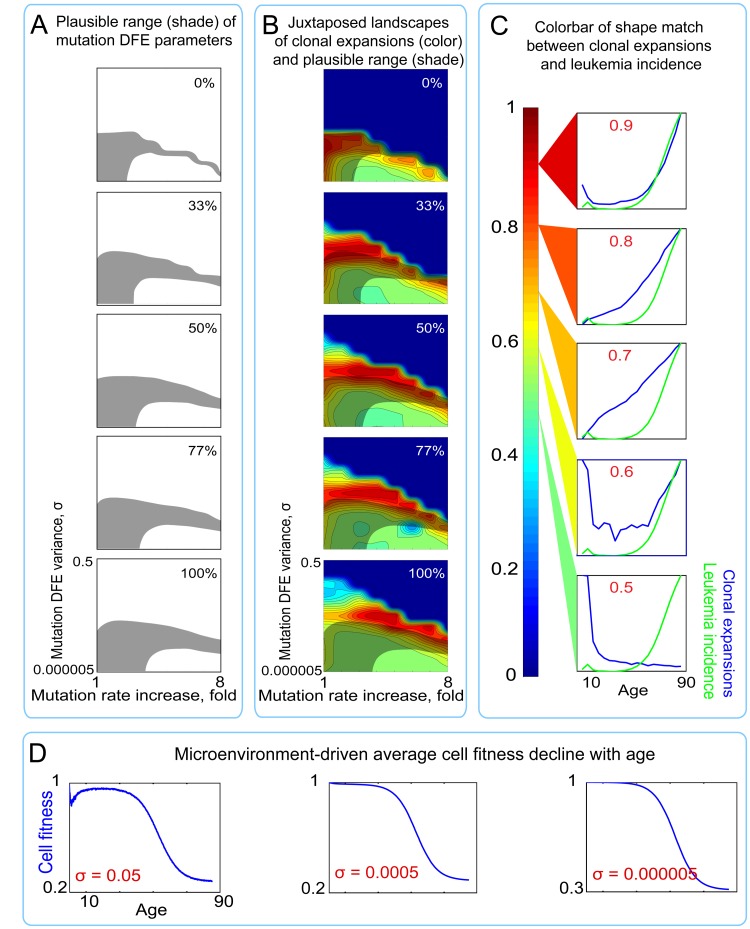
Landscapes of clonal expansions in the simulated pools under different parameters of mutation DFE when cell fitness is modified by microenvironment (**A**) Shaded regions represent the plausible range of mutation DFE variance and rate increase over lifetime (for derivation see Fig. 4) under different proportions of mutations in the positive tail of the ENV DFE (**B**) Plots of shape match landscapes within the studied ranges of mutation DFE variance (σ; Y-axis) and mutation rate increase (X-axis). Colored landscapes represent age-dependent rates of somatic evolution as shown in the panel C. The proportion of ENV DFE variance in the positive tail is indicated in white text. Plausible ranges of mutation parameters from panel A are compared to mutation parameters that replicate exponentially increasing rates of somatic evolution that shape-match leukemia incidence curve. (**C**) Color scale for panel B; colors represent the goodness of shape match between age-dependent leukemia incidence (green line) and simulated clonal expansions (blue line depicts the share of the most successful clone at any given time). (**D**) Age-dependent average cell fitness decline in the simulated pool for the indicated values of mutation DFE (σ).

We applied this additional bicomponent modification of mutation fitness effects by microenvironment and tested the same series of outputs in a composite model, varying the proportion of the microenvironment-imposed DFE (ENV DFE) that resides in the positive tail. The composite model results in an extensive overlap between the *plausible range* (shaded areas in Fig. 8A,B) and conditions allowing for exponential age-dependent increase in clonal expansions (red areas in Fig. 8B; see color bar in Fig. 8C). Moreover, the magnitudes of these clonal expansions are substantially higher in the composite versus the mutations-only model (Fig. 6; Table S1). Both the extent of overlap and the magnitude of clonal expansions are maximal for a small (0-1%) positive tail for microenvironment-imposed DFE. As shown in Fig. 8D, the overwhelming cumulative effect of the microenvironmental uniform component results in the expected substantial average fitness drop, but without promoting excessive purifying selection to buffer fitness decline. In this way, the bi-component model of tissue microenvironment effects, by interacting with the mutation pre-generated cellular diversity, creates a range of mutation DFE variance and rate increase that are concurrently permissive for realistic mutation accumulation, exponential age-dependent increase in rates of somatic evolution (clonal expansions), and realistic cell fitness decline.

Given different estimates of the size of adult human HSC pool, we also tested if the results shown in Fig. 5 and 8 are sensitive to pool size. Both with a larger HSC pool kept at a stable size through adult ages and a pool size increasing over the whole life, the composite mutation/microenvironment model demonstrates a much greater overlap between DFE parameters that replicate leukemia incidence-like clonal expansions in the simulated pool and the slope of mutation accumulation in the Tier 3 genome (Fig. 6B and S3; Table S1B), suggesting that the principal discrepancy between the mutations-only (Fig. 5B) and composite (Fig. 8B) models is independent of pool size. We also tested the same model output under a lower adult cell cycling speed, as the lowest published estimates based on telomere attrition dynamics suggest ~0.6 divisions per year for human HSC [21]. The results in Fig. 6B and Table S1 demonstrate that the discrepancy in the mutations-only model still persists and is corrected by the composite model.

## DISCUSSION

Our results indicate that parameters of the mutation process that would concomitantly allow for exponentially increasing rates of somatic evolution (incidence of leukemia) and significant functional decline in HSC, with both being driven and rate-limited by cell-intrinsic genetic damage accumulation with age, are unlikely to exist. Our modeling instead indicates that a common set of mutation DFE and rate parameters can only be defined under the dynamic, environment-dependent paradigm of fitness. Thus, these results argue that the late life increased incidence of hematopoietic malignancies is not rate-limited by the occurrence of oncogenic genetic damage events, but results from microenvironment-imposed increases in positive selection for previously accumulated genetic/phenotypic diversity in aged tissues.

Our model applies the definition of mutation fitness effects on somatic cells based on Shelford's and Sprengel-Liebig's laws, and operates with a bicomponent evolutionary pressure on HSCs: a uniform pressure on cellular fitness that causes substantial HSC fitness decline with age, as well as a distributed component that interacts with the phenotypic cell diversity generated by mutations and creates a context-dependent fitness differential in the HSC pool. The Sprengel-Liebig system can also be considered bicomponent and roughly holds that deviation of multiple environmental factors from the optimum lowers general population fitness and promotes selection for the better fit; the exact degree and mode of its action on any particular phenotype, however, varies depending on the phenotype and the degree of deviation of particular environmental factors. For example, a mutation conferring resistance to hypoxia could be non-adaptive or neutral in a normoxic tissue, while clearly adaptive under hypoxia (with the magnitude of the effect being dependent on the extent of hypoxia). Similarly, while oncogenic mutations may have a defined phenotypic effect on the recipient cell, the fitness/selective value of the physiological changes they confer will always depend on microenvironment. Our model thus proposes a radical revision of the long-held concept of defined fitness effects of oncogenic mutations in the modeling of cancer with age. Instead, fitness is ultimately a property imposed and defined by microenvironment, and changes in accord with alterations of the microenvironment.

Our model also indicates that the ability of mutations to improve cell fitness should be limited in the young compared to the elderly. Whether young stem cells reside at a local fitness optimum remains an open question, however active selection at the population level over long evolutionary periods should have driven co-evolution of stem cells and young tissue microenvironments to optimize performance. In an aged microenvironment of a post-reproductive animal, cellular adaptation capacity is limited to somatic evolutionary processes, as selection at the germline level is relaxed. In this way, our model is consistent with modern evolutionary models of aging, which explain animal lifespan diversity by evolution of different strategies for age-dependent investment in the maintenance of tissue architecture and function [28, 29, 65]. The idea that this investment is regulated above the cell-intrinsic level is supported by ample evidence of drastically different lifespans within physiologically close groups of mammals, with examples of even significant inter-population lifespan divergence within the same species [66]. This strategy is manifested in the curve of age-dependent microenvironmental effects on cell fitness, with the maintenance of tissue fitness through reproductive years followed by a gradual decline (Fig. 7B).

Likewise, the model can explain the universal age-dependent pattern of cancer incidence as rate-limited primarily by microenvironmental effects that suppress or promote somatic evolution in an age-dependent manner, as dictated by differential investment in tissue maintenance evolved for particular species. This explanation is also consistent with scaling of cancer incidence and aging dynamics to the lifespans of different species. Along with a number of other proposed mechanisms [31], suppression of somatic evolution by age-dependent investment in tissue maintenance could explain why large and long-lived mammals with supposedly larger stem cell pools, and thus seemingly greater chances to acquire cancer driver mutations over longer periods of life, are not more prone to develop cancer compared to smaller, short-lived species [24].

In agreement with evolutionary models of aging, our results suggest that cancer is not rate-limited by the occurrence of oncogenic mutations as postulated by the current model of carcinogenesis, but its incidence instead is tightly linked to the evolution of lifespan and is promoted by altered selective value of oncogenic mutations that accumulate over lifetime in aged tissues. From this perspective, aging is not only the main prognostic factor of cancer rates, but is a factor directly promoting somatic evolution and cancer. Our results, thus, are concordant with other studies demonstrating the impact of aged tissue environments on stem cell fitness [67-70], and the importance of tissue microenvironment during tumor progression [22, 41-46]. It should be noted, however, that besides modifying the fitness of a phenotype, microenvironmental degradation itself is likely to induce additional somatically heritable changes in cells (e.g. epigenetic changes). Empirical evidence indicates that at least to some degree aged phenotypes in HSC are maintained even when removed from the aged tissue microenvironment, such as in *in vitro* culture or following transfer of cells into young recipient mice [71].

Our model incorporates fundamental properties of the evolutionary process (such as context-dependent fitness) with known properties of human HSC pools, and thereby resolves many of the discrepancies underlying previous cancer models, which have hindered our understanding of the relationship between aging and cancer. The utility of our model lies in its ability to integrate into a complex and dynamic system a number of processes and characteristics of stem cells that change non-linearly with age, such as pool size, cell division rates or clonal dynamics. This capability of the stochastic approach has a critical advantage over traditional analytical models used in modeling cancer by providing a tool to describe complex multi-factorial and non-linear processes which may not have analytical solutions. Importantly, our computational model is based on infinitely adaptable parameters (such as pool size, mutation accumulation kinetics, and cell division rates) which can be updated as new experimental data are reported, and adapted to replicate the dynamics of other stem cell systems, thereby allowing for versatile modeling of a myriad of cancer types.

A full appreciation of the critical role for altered selection in cancer development could significantly impact treatment and prevention strategies by shifting focus toward microenvironmental factors modulating cancer evolution, both for initial tumor development and following therapy. Identifying and preventing age-related processes in tissue microenvironments, such as by modulating chronic inflammation or the accumulation of particular byproduct metabolites that could be key in promoting somatic evolution, might prove effective in reducing cancer risk. Cancer therapeutic strategies, such as targeted therapies, might also benefit by shifting focus from inhibition of malignant cell phenotypes to suppressing cell fitness, which requires identification and consideration of specific factors in the microenvironment that affect the fitness value of particular cellular phenotypes. In this regard, analyses of the changes in tissue adaptive landscapes post-treatment could prove helpful in eliminating the potential of pre-treatment tumors to evolve drug-escaping cellular phenotypes within the genetic background of the specific initial tumors.

## SUPPLEMENTAL METHODS FIGURES AND TABLES


