# Supplementary material for: Stochastic modeling indicates that aging and somatic evolution in the hematopoietic system are driven by non-cell-autonomous processes
Source: Aging (Albany NY). 2014 Dec 17;6(12):1033–48. doi: 10.18632/aging.100707 (PMC4298364; doi:10.18632/aging.100707)
Supplement: Supplementary file 1 [file aging-06-1033-s001.docx]

**SUPPLEMENTAL METHODS**

**Model architecture**

Both functional and modeling studies have estimated that there are approximately 11,000 HSC in an adult human, with around 300 HSC present in a newborn human [[1](#_ENREF_1), [2](#_ENREF_2)], with these numbers being limited by the niche space capacity. Thus, our model operates with a matrix of an initial 300 simulated HSC expanding by division up to the adult pool size of 11,000 cells. The simulated pool size changes over lifetime as defined by the function:

$$C_{t}=C_{t-1}+0.008\times\left(C_{t-1}\times\left(\frac{C_{max }- C_{t-1}}{C_{max}}\right)\right)$$

where C_0_ is the initial pool capacity of 300 cells, C_t_ denotes pool capacity at time *t* (in weeks) rounded to the nearest integer, and C_max_ represents the maximum pool capacity of 11,000 cells. The maximum pool size is reached approximately at the age of 18-20 years and is kept stable throughout adult life (**Fig. S1A**).

Each simulated cell in the pool has the following properties: clonal identifier, time since last division, number of point mutations in Tier 3 of the genome, and cell fitness. The model simulates cell dynamics in HSC pools throughout 85 years (4420 weeks) and is updated in weekly increments, whereby cells divide, acquire DNA mutations, change their fitness, or leave the pool (to simulate death, differentiation or senescence) based on the algorithm of cell decisions shown in **Fig. 2**.

The average frequency of cell divisions at any given age was inferred from published data [[2-4](#_ENREF_2)], which demonstrate much more rapid cell cycling in early childhood relative to adulthood, leveling off in adults at an average HSC division frequency of once per 40-45 weeks (ranging from ~25 to over 60 weeks). A similar rapid slowdown in HSC cycling post-maturation has been reported in mice [[5](#_ENREF_5)], suggesting that this pattern may be universal in mammals. Thus, we adopted the adult cycle rates that level off at about one cell division per 40 weeks with a standard deviation of 5 weeks (**Fig. S1B**). The decision of a cell to divide is made by comparing its time past last division to a random number drawn from a normal distribution having the mean,µ, equal to the current average cycling speed and a standard deviation σ = µ/8. We thus kept variance proportional to the current mean, the ratio being inferred based on published average rates and variance [[2](#_ENREF_2), [3](#_ENREF_3)]. After each division the *time past division* parameter was reset to 1 (week).

At each division, point mutations were added based on the mutation rate per bp and the size of the Tier 3 genome estimated at 1,182,046,340 bp [[6](#_ENREF_6)]. Each dividing cell thus accumulated an additional *M*  x 1,182,046,340 Tier 3 mutations per division, where *M* stands for point mutation rate per bp per cell division. The mutation rate replicated by the model is consistent with ca. 3 x 10^-9^ per bp per cell division, which agrees with published estimates [[7](#_ENREF_7), [8](#_ENREF_8)].

The key functional parameter that changed upon cell division was *cell fitness*, initially set to 1 for all cells (at “birth”). The model integrates all types of cell-intrinsic random DNA alterations (like DNA mutations and epigenetic changes, which we hereby combine under the term *mutations*) into one process based on the assumption that there exists a combined DFE of all possible forms of DNA damage per cell division. We excluded the lethal portion of the DFE from the model, assuming that lethal mutations are equally likely to occur in all cells and do not affect somatic evolution. The rest of the DFE is represented with mostly neutral mutations based on a composite normal distribution with its mode equal to 0 and tails of different relative sizes (**Fig. S1D**). We asked how the shape and variance of this DFE affects somatic evolution in the simulated pool by using a number of possible tail ratios and a plausible range of variance expressed as standard deviation, σ.

An increasing mutation rate over lifetime would increase the total impact on cell fitness. We used the rule of distribution sums [[9](#_ENREF_9)] to accommodate the modeled mutation DFE per cell division to an increasing mutation rate over lifetime. As shown in **Fig. S1E-F**, an increasing mutation rate was modeled by increasing the variance of the net mutation DFE per cell division proportionally to the increase of mutation rate.

We used whole genome sequencing (WGS) mutation accumulation data from the Tier 3 of AML genomes and DNA methylation accumulation in the hematopoietic cells (**Fig. 3A,B**; [[10](#_ENREF_10), [11](#_ENREF_11)]) as a reference for the natural slope of mutation accumulation in HSC genomes. These data closely resemble estimates from another study [[7](#_ENREF_7)], suggesting that roughly a third to half of total Tier 3 mutations accumulate by the age of 20, slowing down thereafter to reach about 500-600 mutations per cell over a lifetime. Tier 3 mutation accumulation in pediatric acute megakaryoblastic leukemia (AMKL, a subset of AMLs) demonstrates a much more rapid increase in mutation accumulation in the first few years of life [[12](#_ENREF_12)], likely due to much faster cell cycling rates. As argued by others [[7](#_ENREF_7)], mutation accumulation in AML should reflect mutation accumulation in individual HSC, given that AML initiates in HSC and thus should capture Tier 3 passenger mutations present in the initiated HSC. Although differences may exist between normal and AML cells in cycling or mutation rates, these should not affect the slope of mutation accumulation in non-selected genome tiers, given that any such bias should be similar at different ages. A recent study analyzed DNA mutations in normal human early hematopoietic progenitor cells by exome sequencing of individual progenitor cells [[7](#_ENREF_7)], but the very small sample size analyzed in that study does not provide sufficient power to determine substitution rates with age. Moreover, unlike the Tier 3, exomic mutations often have phenotypic effects and thus are subject to selection which could distort the resulting accumulation curve.

The model considers fitness as a measure of a cell’s ability to expand its progeny in the pool, which is reflected in the ratio of the cell cycling speed to its likelihood to leave the competitive pool by differentiating, dying or senescing. We integrate these properties in two parameters, *cell cycling speed* and a cell’s *likelihood to leave the pool* for whatever reason. Cell fitness differential, and thus competition, is realized through different probabilities for cells to leave the pool. At each “weekly” update of the HSC pool state, cells are tested in a binomial trial to decide between staying and leaving the pool based on the probability calculated as follows:

$\text{P}_{\text{i}}=\frac{\text{C}_{\text{t}}\times\text{F}_{\text{i}}}{\sum_{\text{k}=1}^{\text{N}} \text{F}_{\text{k}}}$ (3)

where Pi stands for the probability of the i^th^ cell to stay in the pool at time *t* in a binomial trial X ~ B(1, P_i_); N is the current number of cells after all cell divisions, C_t_ represents the pool capacity at time *t*, and F_i_ is the i^th^ cell’s fitness. In this way, a given cell’s probability to remain in the pool at each trial depends on how many cells compete for the current space and the cell’s fitness relative to other cells.

Finally, the model replicates cell divergence through division and functional/fitness change over time by designating groups of cells with similar fitness derived from common predecessors as clones. At the simulation start, a unique clonal ID is randomly generated from the uniform distribution X ~ unif(2, 99999) and assigned to each cell. This ID is inherited by all the cell’s descendants except cells that change their fitness during one division more than a certain threshold value, this threshold being called the *clonal scale*. Cells changing their fitness during one division by more than the clonal scale threshold were assigned a separate clonal ID, initiating thus a new clone. We expressed this scale as the number of σ’s of the initial mutation DFE. As shown in **Fig. S1C**, the clonal scale affects the total clonal diversity in the pool and uncertainty in the model’s behavior. As such, clonal scale is a measure of the magnitude of fitness change during one cell division that makes the incipient cell a separate clone and thus allows tracking its progeny’s success in competition. In this way, new clones that appear over time in the course of simulation essentially represent cells bearing a mutation with a significant phenotypic effect. We adopted the clonal scale equal to 3.5 σ’s of the mutation DFE, which allowed slightly over 20% of uncertainty in the outcome and with a maximum of about 2000 distinct clones in the pool. Thus, the clonal scale is a reasonably chosen but arbitrary value, because functional divergence in nature is a continuous process.

**Model output**

We measured three main types of model output. As Tier 3 mutation accumulation had two phases, a quick accumulation from birth till adulthood followed by a slower phase throughout the adult life, *mutation accumulation slope* was measured by linear regression of the segment from the ages of 2000 weeks (~38 years) to 4000 weeks (~77 years).

We also registered the share (%) of the most expanded/successful clone in the pool at each weekly simulation update to track the dynamics of clonal expansions over the simulated lifetime. To match the resulting curve with leukemia incidence, we first normalized both leukemia and the resulting clonal expansions curve by 0-1 normalization, so that we were able to negate the effect of scale and compare shapes. The clonal expansions curve was approximated to 19 time/age points to match the leukemia reference data which are averaged in 5 year increments to make 19 age points. The shape comparison was done by the Mean Root Square Error (MRSE) method, whereby the shape match was calculated by subtracting the MRSE difference between curves from 1 (i.e. 1-MRSE). This method resulted in the similarity measure from 0 (no resemblance) to 1 (perfect curve shape match). Thus the second output measured was the age-dependent *curve similarity* between leukemia incidence and simulated clonal expansions.

And lastly, we measured the percent of pool occupied by the most successful clone at the end of the simulated life to infer the maximum fitness gain possible under a given mutation DFE parameters over lifetime. Thus, we asked if a given combination of mutation DFE parameters allowed expansion or suppression of clonal expansions over the simulated lifespan. The value of this terminal clonal expansion was used to infer the *magnitude of clonal expansions* possible under a given set of mutation DFE parameters.

All the three types of output were registered for a range of possible combinations of mutation DFE variance (expressed in standard deviations, σ), mutation rate increase over lifetime (expressed in fold increase over the initial rate), and tail ratios of mutation DFE (expressed in the percent of DFE variance in the positive tail of the distribution).

Initially, registration of a given output was done by building a two-dimensional matrix of output values as shown in **Table S2**.

This initial 11x8 matrix was then transformed into a larger 1001x701 matrix with smaller increments along both dimensions by interpolating intermediate values using the Matlab spline function. This transformation resulted in a large matrix allowing smooth surfaces of the initial output values to be built.

Each output value in the initial table shown above was calculated as the average of 6 simulated lifespans (individuals). However, after spline-based transformation into a large 1001x701 matrix, each output value in the matrix was in fact corrected by the spline regression algorithm against all neighboring points, which significantly increased the effective “sample size” for each output value from the initial 6 measurements. Therefore, given the general ~20% uncertainty in the model output set by the chosen *clonal scale* (explained in the model architecture part of the main text), the effectively large underlying sample size for each point output values makes the resulting surface reliable.

For the initial output matrix shown in the example table above, we used linearly spaced values of mutation rate fold increase over lifetime from stable rate (1-fold) to 8 fold.

We spaced mutation DFE variance values (σ) exponentially between 0.5 and 0.000005 so that their Log_10_ values were distributed linearly within the range. The Log_10_ scaling was done to linearize the spacing of the measured σ values within the studied range based on their expected effect on the output variable. Based on the properties of normal distributions, the percent of values equal or greater than any given value within the distribution is defined by variance. For instance, values above ± 2σ represent roughly 5% of the total distribution. In distributions of fitness effects, therefore, a change in σ that results in a relative fitness change of a given magnitude is proportional to the DFE σ itself. In other words, DFEs with greater σ (wider distributions) require a larger absolute change in σ to achieve the same relative change in fitness compared to narrow DFEs. Due to this property, the actual changes in the fitness effects drawn from a DFE within a given range of DFE σ are only linearly spaced on the logarithmic scale.

**SUPPLEMENTAL FIGURES AND TABLES**


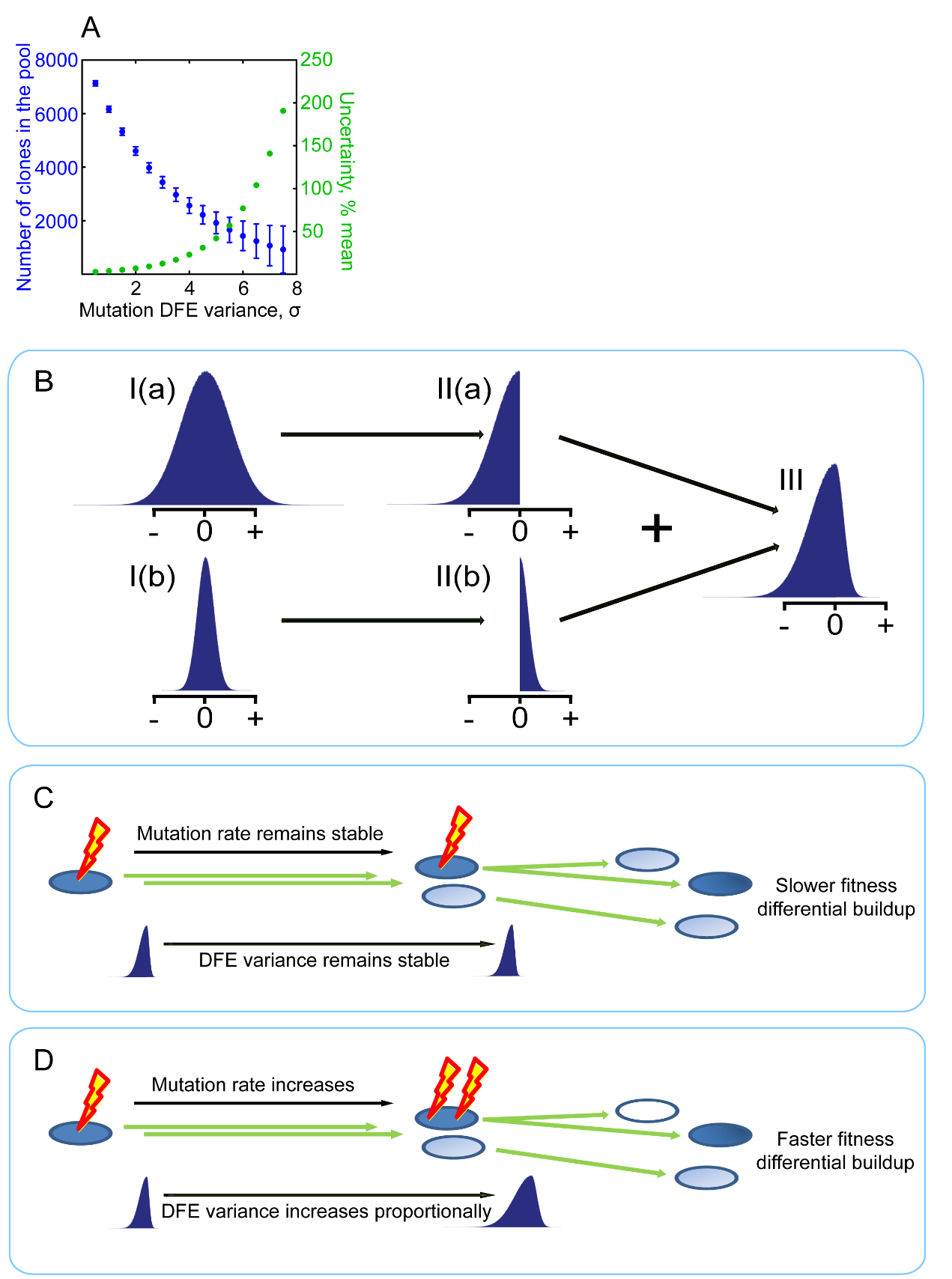


**Figure S1. Key model parameters.** **(A)** The minimum amount of cell fitness change per division for a cell to be assigned a separate clonal status (clonal scale; expressed in standard deviations of the mutation DFE) affects uncertainty in model behavior and the number of clones in the pool). **(B)** Mutation DFE with unequal tails is generated by adding two numbers drawn from Gaussian distributions with different variances (I(a) and II(a)), whereby one number is made negative (II(a)) and the other positive (II(b)) based on desired tail proportions of the combined DFE. **(C)** Stable mutation rate: a cell’s fitness change per division is drawn from the same DFE over lifetime. **(D)** Increasing mutation rate with age: a cell’s fitness change per division is drawn from a DFE with variance increasing proportionally to the mutation rate over lifetime based of the law of probability sums.


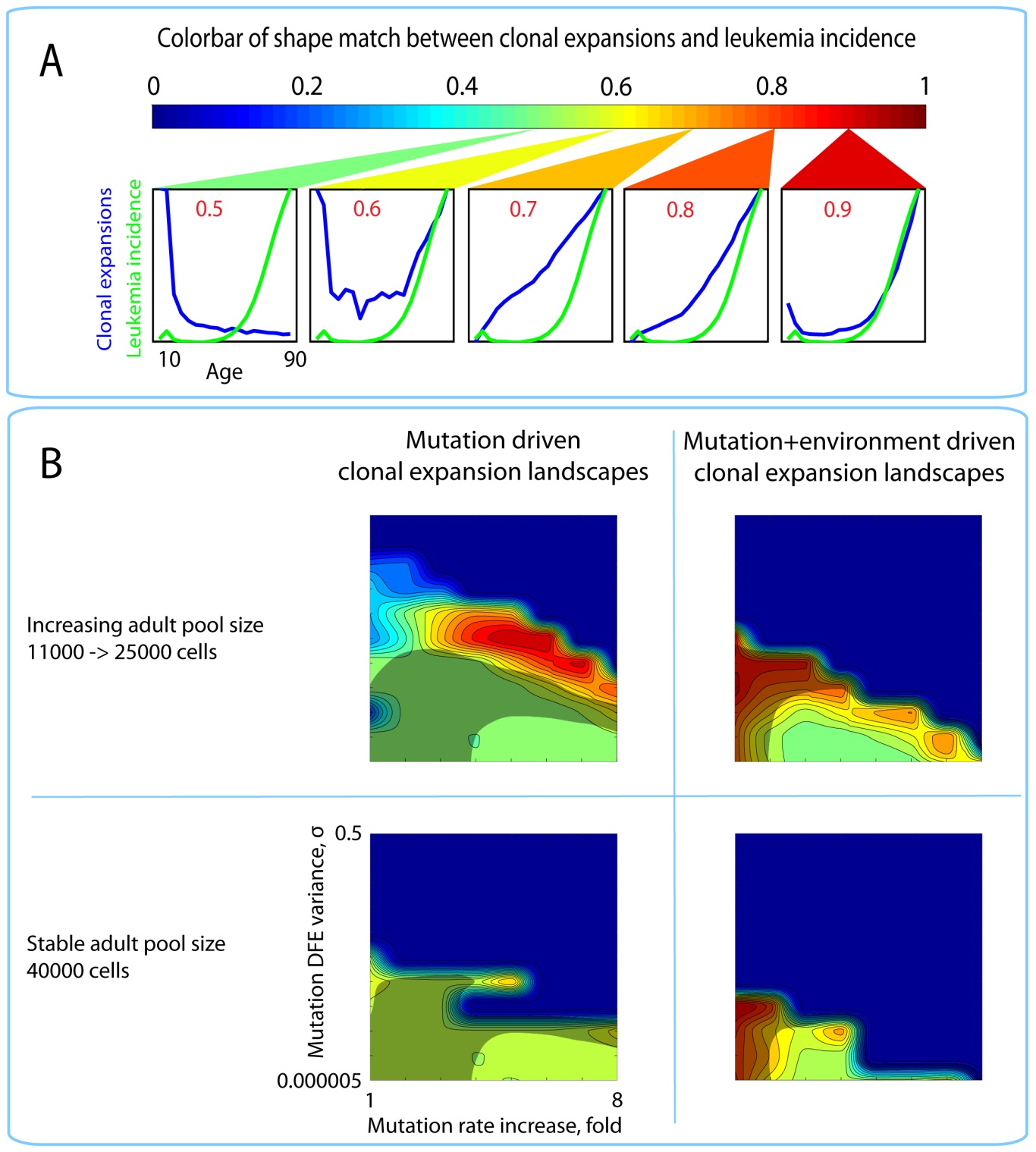


**Figure S2. Landscapes of clonal expansions in the simulated pools including the plausible range for the slope of DNA methylation accumulation with age. (A)** Color bar for panel B; colors represent the goodness of shape match between the age-dependent leukemia incidence (green line in panel A charts) and simulated clonal expansions (blue line in panel A charts). **(B)** Plots of shape match landscapes within the studied ranges of mutation DFE (left) and microenvironmental DFE (right) parameters. Shaded areas in each plot represent mutation (left) or mutation+environment DFE (right) parameter ranges under which the simulated age-dependent slope of mutation accumulation in Tier 3 genome is within the 95% confidence interval of the reference slope for AML genomes. The slope for DNA methylation accumulation was derived based on data from Horvath (2013) (see Fig. 3B). The percent of mutation DFE in the positive tail in all plots is 1% and for microenvironmental DFE is 0%.


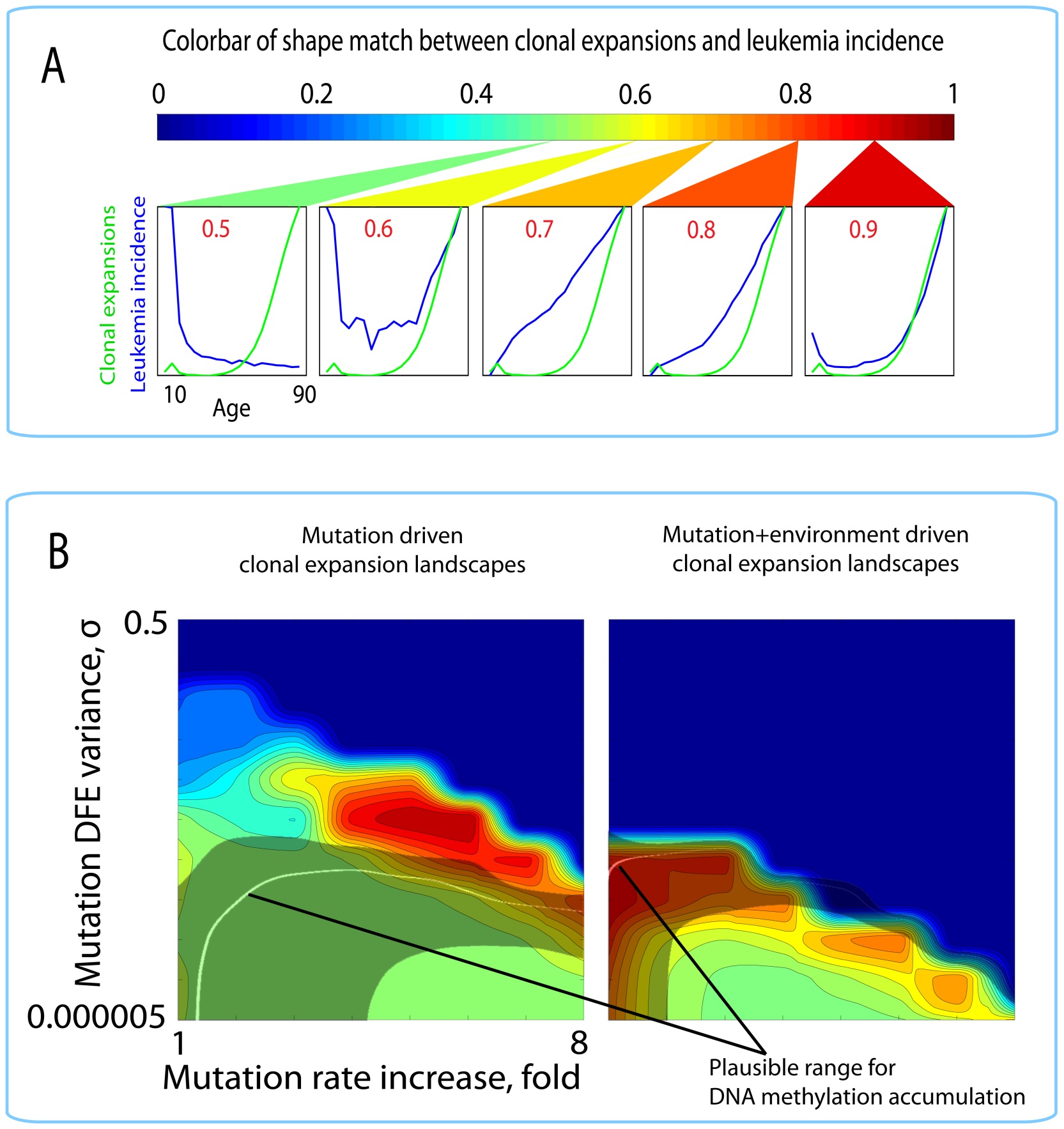


**Figure S3. Landscapes of clonal expansions in the simulated pools of different sizes under different parameters of mutation DFE and microenvironment.** **(A)** Color bar for panel B; colors represent the goodness of shape match between the age-dependent leukemia incidence (green line in panel A charts) and simulated clonal expansions (blue line in panel A charts). **(B)** Plots of shape match landscapes within the studied ranges of mutation DFE (left) and microenvironmental DFE (right) parameters. Shaded areas in each plot represent mutation (left) or mutation+environment DFE (right) parameter ranges under which the simulated age-dependent slope of mutation accumulation in Tier 3 genome is within the 95% confidence interval of the reference slope for AML genomes. The percent of mutation DFE in the positive tail in all plots is 1% and in microenvironmental DFE is 0%.

| 1. **Simulated adult pool size kept stable at 11,000 cells** | | | | | | | |
| --- | --- | --- | --- | --- | --- | --- | --- |
| **Mutations only** | **Stringency** | **Tested parameters** | **% of mutation DFE in the positive tail** | | | | |
|  | **% mutation DFE in positive tail** | | **0.00** | **1.00** | **10.00** | **33.00** | **50.00** |
|  | **0.7** | overlap | 4.95 | 5.56 | 0.15 | 0.00 | 0.00 |
|  |  | average expansion, % pool | 4.35 | 4.08 | 3.25 | 0.00 | 0.00 |
|  | **0.8** | overlap | 0.55 | 0.94 | 0.00 | 0.00 | 0.00 |
|  |  | average expansion, % pool | 7.97 | 6.13 | 0.00 | 0.00 | 0.00 |
|  | **0.9** | overlap | 0.00 | 0.00 | 0.00 | 0.00 | 0.00 |
|  |  | average expansion, % pool | 0.00 | 0.00 | 0.00 | 0.00 | 0.00 |
| **Mutations + environment** | **Stringency** | **Tested parameters** | **0% of CAD DFE in the positive tail** | | | | |
|  | **% environmental DFE in positive tail** | | **0.00** | **33.00** | **50.00** | **77.00** | **100** |
|  | **0.7** | overlap | 42.48 | 25.83 | 27.27 | 16.43 | 10.40 |
|  |  | average expansion, % pool | 21.89 | 8.73 | 8.17 | 5.70 | 5.85 |
|  | **0.8** | overlap | 24.80 | 13.11 | 12.51 | 4.14 | 4.14 |
|  |  | average expansion, % pool | 28.18 | 11.68 | 13.01 | 9.44 | 8.86 |
|  | **0.9** | overlap | 1.60 | 1.91 | 0.25 | 0.00 | 0.00 |
|  |  | average expansion, % pool | 17.38 | 18.59 | 14.93 | 0.00 | 0.00 |
| 1. **Comparison of model performance under different adult pool sizes** | | | | | | | |
| **Mutations only** | **Stringency** | **Tested parameters** | **1% of mutation DFE and 0% of environmental DFE in the positive tail** | | | | |
|  | **Adult HSC pool size, cells** | | **stable, 11000** | **increasing, 11000 -> 25000** | **stable, 40000** | **slower cell cycling, 11000** | |
|  | **0.7** | overlap | 5.56 | 0.01 | 0.00 | 0.00 | |
|  |  | average expansion, % pool | 4.08 | 2.85 | 0.00 | 0.00 | |
|  | **0.8** | overlap | 0.94 | 0.00 | 0.00 | 0.00 | |
|  |  | average expansion, % pool | 6.13 | 0.00 | 0.00 | 0.00 | |
|  | **0.9** | overlap | 0.00 | 0.00 | 0.00 | 0.00 | |
|  |  | average expansion, % pool | 0.00 | 0.00 | 0.00 | 0.00 | |
| **Mutations + environment** | **0.7** | overlap | 49.40 | 49.99 | 46.15 | 50.24 | |
|  |  | average expansion, % pool | 23.91 | 24.16 | 2.67 | 14.03 | |
|  | **0.8** | overlap | 34.94 | 30.72 | 19.43 | 18.87 | |
|  |  | average expansion, % pool | 29.70 | 33.51 | 4.12 | 18.88 | |
|  | **0.9** | overlap | 0.09 | 0.62 | 2.65 | 0.14 | |
|  |  | average expansion, % pool | 65.43 | 22.55 | 6.48 | 11.93 | |

**Table S1. Clonal dynamics in simulated pools under different parameters of the mutation DFE, microenvironment DFE, and adult pool sizes.** *Overlap*: percent of overlap between DFE parameters that replicate exponential age-dependent clonal expansions in the simulated pool and the slope of mutation accumulation in Tier 3 AML genomes. *Average expansion*: average percent of the pool occupied by the most successful clone within the *overlap*. *Stringency*: a minimum shape match threshold between the simulated age-dependent clonal expansions and the age-dependent leukemia incidence measured by MSRE from 0 (no match) to 1 (perfect match) to delineate the range of DFE parameters that replicate exponential age-dependent clonal expansions in the simulated pool (see Fig. 5A for a visual guide).

|  | **Mutation rate increase over lifetime, x fold** | | | | | | | |
| --- | --- | --- | --- | --- | --- | --- | --- | --- |
| **Variance of mutation DFE,** σ | **1** | **2** | **3** | **4** | **5** | **6** | **7** | **8** |
| **0.5** | 0 | 0 | 0 | 0 | 0 | 0 | 0 | 0 |
| **0.158114** | 0 | 0 | 0 | 0 | 0 | 0 | 0 | 0 |
| **0.05** | 0.332 | 0.478 | 0.000 | 0.000 | 0.000 | 0.000 | 0.000 | 0.000 |
| **0.015811** | 0.303 | 0.291 | 0.33774 | 0.391 | 0.000 | 0.000 | 0.000 | 0.000 |
| **0.005** | 0.384 | 0.554 | 0.682 | 0.705 | 0.721 | 0.000 | 0.000 | 0.000 |
| **0.001581** | 0.458 | 0.39329 | 0.679 | 0.903 | 0.960 | 0.955 | 0.000 | 0.000 |
| **0.0005** | 0.540 | 0.533 | 0.522 | 0.584 | 0.737 | 0.853 | 0.907 | 0.000 |
| **0.000158** | 0.565 | 0.532 | 0.516 | 0.515 | 0.541 | 0.591 | 0.754 | 0.820 |
| **0.00005** | 0.563 | 0.527 | 0.517 | 0.525 | 0.520 | 0.543 | 0.570 | 0.675 |
| **1.58E-05** | 0.56501 | 0.526 | 0.517 | 0.519 | 0.516 | 0.528 | 0.538 | 0.561 |
| **5E-06** | 0.56322 | 0.52652 | 0.520 | 0.512 | 0.523 | 0.529 | 0.533 | 0.542 |

**Table S2. An example of the initial matrix of model output within the *mutation DFE variance* and *mutation rate increase over life-time* parameter space.** The indicated combinations of the two parameters were used to run the model and the output was plotted for each combination. In this example the output values represent shape similarity between the age-dependent curve of clonal expansions (extent of somatic evolution) and the age-dependent curve of leukemia incidence. The similarity was measured by the MSRE method and ranges from 0 (no similarity) to 1 (perfect shape match).

**SUPPLIMENTAL REFERENCES**

1. Abkowitz JL, Catlin SN, McCallie MT and Guttorp P. Evidence that the number of hematopoietic stem cells per animal is conserved in mammals. Blood. 2002; 100(7):2665-2667.

2. Catlin SN, Busque L, Gale RE, Guttorp P and Abkowitz JL. The replication rate of human hematopoietic stem cells in vivo. Blood. 2011; 117(17):4460-4466.

3. Shepherd BE, Guttorp P, Lansdorp PM and Abkowitz JL. Estimating human hematopoietic stem cell kinetics using granulocyte telomere lengths. Exp Hematol. 2004; 32(11):1040-1050.

4. Sidorov I, Kimura M, Yashin A and Aviv A. Leukocyte telomere dynamics and human hematopoietic stem cell kinetics during somatic growth. Exp Hematol. 2009; 37(4):514-524.

5. Bowie MB, McKnight KD, Kent DG, McCaffrey L, Hoodless PA and Eaves CJ. Hematopoietic stem cells proliferate until after birth and show a reversible phase-specific engraftment defect. J Clin Invest. 2006; 116(10):2808-2816.

6. Mardis ER, Ding L, Dooling DJ, Larson DE, McLellan MD, Chen K, Koboldt DC, Fulton RS, Delehaunty KD, McGrath SD, Fulton LA, Locke DP, Magrini VJ, et al. Recurring mutations found by sequencing an acute myeloid leukemia genome. N Engl J Med. 2009; 361(11):1058-1066.

7. Welch JS, Ley TJ, Link DC, Miller CA, Larson DE, Koboldt DC, Wartman LD, Lamprecht TL, Liu F, Xia J, Kandoth C, Fulton RS, McLellan MD, et al. The origin and evolution of mutations in acute myeloid leukemia. Cell. 2012; 150(2):264-278.

8. Lynch M. Evolution of the mutation rate. Trends in Genetics. 2010; 26(8):345-352.

9. Grinstead CM and Snell JL. (1997). Introduction to Probability. (Providence: American Mathematical Society).

10. network Tcgar. Genomic and epigenomic landscapes of adult de novo acute myeloid leukemia. N Engl J Med. 2013; 368(22):2059-2074.

11. Horvath S. DNA methylation age of human tissues and cell types. Genome Biol. 2013; 14(10):R115.

12. Gruber TA, Larson Gedman A, Zhang J, Koss CS, Marada S, Ta HQ, Chen SC, Su X, Ogden SK, Dang J, Wu G, Gupta V, Andersson AK, et al. An Inv(16)(p13.3q24.3)-encoded CBFA2T3-GLIS2 fusion protein defines an aggressive subtype of pediatric acute megakaryoblastic leukemia. Cancer Cell. 2012; 22(5):683-697.

**MATLAB CODES**

**Model code:**

clearvars -except

%model increment = 1 week

% initial parameters:

patients = 1; % number of simulated individuals

%normalized reference curve

leukem = [0.021509723; 0.07279198; 0.017064227; 0.005662811; 0.00473834;...

0.000800442; 0; 0.006614843; 0.013586841; 0.028451863; 0.055538266;...

0.10117379; 0.165714443; 0.267223569; 0.403955584; 0.565278507;...

0.729978904; 0.876156593; 1];

% constant for all patients

maxPool = 11000; % maximum capacity of the pool (max number of HSC)

maxAge = 4420; % maximum age (85 years)

initHSC = 300; %round(300*(maxPool/11000)); % initial number of HSC cells

ageIncrement = 1; % increment of age progression in weeks (weekly pool status update)

HSCpoolLength = maxPool + 2000; % not relevant (the size of the pool plus some spare space for pre-generating empty matrices to save time)

mutRate = 3 * 10^-9; % rate of mutation per cell division per base

mutRateFoldIncrease = 8; % mutation rate increase over lifetime

sigma = 5.00E-06; % σ of mutation DFE

maxFitness = 1; % maximum cell fitness

minFitness = 0.3; % minimum cell fitness (at advanced ages)

%genome tiers: (Based on Mardis et al. 2009 study defining three tiers of the genome mutations in which can have functional consequences)

tier1 = 37397019;

tier2 = 246643533;

tier3 = 1182046340;

%///// OUTPUT STORAGE MATRICES

%////////////////////////////////////////////////////////////////////

numCellDividing = []; %a matrix tracking the number of cells dividing at any given time (not working in parallel computing setup)

clonalDivers = []; %a matrix tracking the number of different clones at any given time

averFitn = [];

HSCs = [];

totMut = []; %a matrix tracking the total number of mutations present in the pool

maxClon = [];

posTail = [];

cellDivsCumulative = [];

%////////////////////////////////////////////////////////////////////

%////////////////////////////////////////////////////////////////////

%////////////////////////////////////////////////////////////////////

%////////////////////////////////////////////////////////////////////

%POOL CAPACITY WITH AGE (%-barred code is an alternative for HSC pool size changing over lifetime)

oldCapacity = initHSC;

capacityArr = [initHSC];

for i = 1 : maxAge %i = 1 : 950

%newCapacity = oldCapacity + (round(0.008 * (oldCapacity * (((maxPool/2) - oldCapacity) / (maxPool/2)))));

newCapacity = oldCapacity + (round(0.008 * (oldCapacity * ((maxPool - oldCapacity) / maxPool))));

capacityArr = [capacityArr, newCapacity];

oldCapacity = newCapacity;

end

%increment = round(maxPool/2/(maxAge-1000));

%for i = 951 : maxAge

% capacityArr = [capacityArr, capacityArr(i-1)+increment];

%end

% dynamics of HSC division

divFitAges = [1 : maxAge];

spf = 0.4; % spf corrects cycling rate estimates

HSCdivSpeeds = [3, 3, 21, 34.8, 41.6];

HSCdivAges = [0, 1, 416, 1040, maxAge];

HSCdivSpeeds = HSCdivSpeeds.';

HSCdivAges = HSCdivAges.';

splin = fit(HSCdivAges, HSCdivSpeeds, 'pchipinterp');

divSigma = feval(splin, divFitAges);

%\\\\\\\\\\\\\\\\\\\\\\\\\\\\\\\\\\\\\\

% FITNESS DECLINE CURVE

fitnessArr = (1 - ((1 - minFitness) ./ (1 + 5200*exp(-.0031.*[1:maxAge]))));

% amount of fitness drop per weekly update

derivat(1, 1) = 0;

for i = 2 : maxAge

derivat(1, i) = sqrt((fitnessArr(1, i)-fitnessArr(1, i-1))^2);

end

derivat(1, :) = derivat(1, :);

%---------------------------------

%\\\\\\\\\\\\\\\\\\\\\\\\\\\\\\\\\\\\\\\\\\\\\\\

% A MATRIX OF AVERAGE CELL DIVISION RATES

%//////////////////////////////////////////////////

randDivAges = zeros(maxAge, HSCpoolLength);

for i = 1 : maxAge

randDivAges(i, :) = normrnd(divSigma(i), divSigma(i)/8, 1, HSCpoolLength);

end

%\\\\\\\\\\\\\\\\\\\\\\\\\\\\\\\\\\\\\\\\\\\\\\\\\\\\

fprintf('patient age group birth time \n') % for visualization purposes only

% patient

for patient = 1 : patients % this loop contains the whole run of the program for each individual

cellDivisions = 0;

totSigma = sigma;

cloneScale = totSigma*3.5; % minimum measure of fitness difference that distinguishes clones (a cell has to be different at least by this parameter to be assigned a separate clonal status)

disp(patient)%------------------------------------------- for visualization only

randDivAgesLocal = randDivAges;

%determining the frequency of mutations

affectedGenomeTier = tier3; %tier1 + tier2 + tier3; %negSelGenome + posSelGenome; %the size of functional genome in bp

fidelity = mutRate * affectedGenomeTier; %determining the replication fidelity in this individual

currCapacity = initHSC; %current capacity of the pool

%//////////////////////////////////////////////////////////////////

%memory preallocation by creating an empty matrix for the whole HSC pool

HSCpool = zeros(6, HSCpoolLength); % matrix of HSC cells (updated dynamically)

% loading initial HSC pool

%-- 1. cell IDs

%-- 2. cell ages

%-- 3. cell mutations

%-- 4. fitness

HSCpool(1:4, 1:initHSC) = [ ones(1,initHSC);...

randi([2, 3], 1, initHSC);...

zeros(1,initHSC);...

ones(1,initHSC)];

HSCpool(5, :) = 0;

%\\\\\\\\\\\\\\\\\\\\\\\\\\\\\\\\\\\\\\\\\\\\\\\\\\\\\\\\\\\\\\\

%initial ## mutations

HSCpool(3, 1:initHSC) = normrnd(75, 75/8, 1, initHSC);

% random initial clonal IDs (each cell ID is unique)

HSCpool(1, 1:initHSC) = randi([2 99999], 1, initHSC);

% this loop runs the whole program for a given individual ("patient")

for currentAge = 1 : ageIncrement : maxAge

totSigma = totSigma + (((totSigma*mutRateFoldIncrease) - totSigma)/maxAge);

% current mutation rate

mutRateCurrent = mutRate + ((mutRateFoldIncrease*mutRate - mutRate)/maxAge)*currentAge;

fidelityLocal = mutRateCurrent * affectedGenomeTier; % # Tier 3 mutations added per cell division

% cell divisions

HSCpool(2, HSCpool(2, :) >= randDivAgesLocal(currentAge, :)) = 1; % resets the time past division for cells that divide

newCells = HSCpool(:, HSCpool(2, :) == 1); %new cells

%|||||||||||||||||||||||||||||||||||||||||||||||||||||||||||||||||||

%cellDivisions = cellDivisions + size(newCells, 2);

%cellDivsCumulative(patient, currentAge) = cellDivisions/poolSizeCumulative*100;

%numCellDividing(patient, currentAge) = size(newCells, 2);

%|||||||||||||||||||||||||||||||||||||||||||||||||||||||||||||||||||

zero_iv = find(HSCpool(1, :) == 0); %index vector of zero elements (empty spaces) within the HSC pool matrix

if ~isempty(newCells)

HSCpool(:, zero_iv(1,1:size(newCells, 2))) = newCells; %adding new cells to the pool

end

% all cells compete for available space based on

%relative cell fitness, space available, the curr # cells

w_prob = find(HSCpool(1, :));

n = size(w_prob,2);

w_prob(2, :) = (capacityArr(currentAge) / n) * (n * HSCpool(4, w_prob(1, :)) / sum(HSCpool(4, w_prob(1, :)))); %weighed probabilities

w_prob(2, w_prob(2,:) >= 1) = 1;

w_prob(3, :) = binornd(1, w_prob(2, :)); % all cells compete for free niches

HSCpool(:, w_prob(1, w_prob(3, :) == 0)) = 0; % all lost cells zeroed

w_prob = [];

% =========================================================

% new cells mutate

mutCells = HSCpool(:, HSCpool(2, :) == 1);

mutCells(3, :) = mutCells(3, :) + floor(fidelityLocal) + binornd(1, fidelityLocal - floor(fidelityLocal), 1, size(mutCells, 2));

%///////////////////////////////////////////////////////////////////////////

%/////// MUTATION FITNESS EFFECTS PER CELL DIVISION//////////////////////////////

previousSelAdv = mutCells(4, :);

mutCells(6, :) = ...

- sqrt((normrnd(0, totSigma)*99/100)^2)...

+ sqrt((normrnd(0, totSigma)*1/100)^2);

mutCells(4, :) = mutCells(4, :) + mutCells(6, :);

%///////////////////////////////////////////////////////////////////////////

%///////////////////////////////////////////////////////////////////////////

%clonal divergence

newClones = find(round(mutCells(4, :)./cloneScale) ~= round(previousSelAdv(1, :)/cloneScale));

mutCells(1, newClones) = randi([2 99999], 1, size(newClones, 2));

mutCells(5, newClones) = currentAge;

%///////////////////////////////////////////////////////////////////////////

%/////// MICROENVIRONMENT'S FITNESS EFFECTS PER CELL DIVISION///////////////

HSCpool(4, HSCpool(1, :) > 0) = HSCpool(4, HSCpool(1, :) > 0) - derivat(1, currentAge) ...

- sqrt(normrnd(0, derivat(1, currentAge)/2)^2);

%+ sqrt(normrnd(0, derivat(1, currentAge)/2*1/100)^2);

zero_iv = find(HSCpool(1, :) == 0); %index vector of zero elements

if ~isempty(mutCells)

HSCpool(:, zero_iv(1,1:size(mutCells,2))) = mutCells; %adding new cells to the pool

end

mut_prob = [];

clones = unique(HSCpool(1, HSCpool(1, :) > 0));

% clonal proportions in the pool

clones(2, :) = histc(HSCpool(1, :), clones(1, :));

clones(3, :) = clones(2, : ) ./ sum(clones(2, :)).*100;

clones(4, :) = HSCpool(5, find(unique(HSCpool(1, HSCpool(1, :) > 0))));

%clones = [clones; clonStatus];

oncClones = clones(:, clones(4, :) > 0);

if size(clones, 2) > 0

maxClon(patient, currentAge) = max(clones(3, :));

end

% updating cell ages

HSCpool(2, HSCpool(1, :) ~= 0) = HSCpool(2, HSCpool(1, :) ~= 0) + 1;

%tracking total mutations

currMut = sum(HSCpool(3, :)) / size(find(HSCpool(3, :)), 2);

totMut(patient, currentAge) = currMut;

%TRACKING FITNESS CHANGE

averFitn(patient, currentAge) = mean(HSCpool(4, HSCpool(1, :) > 0));

if currentAge > 1

clonDistrib(patient, currentAge) = max(clones(3, :)) + clonDistrib(patient, currentAge - 1);

else

clonDistrib(patient, currentAge) = max(clones(3, :));

end

clonalDivers(patient, currentAge) = size(unique(HSCpool(1, :)), 2);

HSCs(patients, currentAge) = size(find(HSCpool(1, :)), 2);

% the proportion of cels in the positive tail

%modeFit = mode(HSCpool(4, HSCpool(1, :) > 0));

%numPos = numel(HSCpool(1, HSCpool(4, :) > modeFit));

%posTail(patient, currentAge) = numPos/numel(HSCpool(1, HSCpool(1, :) > 0))*100;

%$$$$$$$$$$$$$$$$$$$$$$$$$$$$$$$$$$$$$$$$$$$$$$$$$$$$$$$$$$$$$$$$$$$$$$$$$$$$$$

end

% MEMORY CLEANUP

newCells = [];

end

%////////////////////////////////////////////////////////

% AVERAGING CLONAL DIVERSITY FROM 6 PATIENTS

%cloneDivAver6 = zeros(1, size(clonalDivers, 2));

%for i = 1 : size(clonalDivers, 2)

% cloneDivAver6(1, i) = mean(clonalDivers(:, i));

%end

%disp('clones 1000-1500:')

%fprintf([num2str(mean(cloneDivAver6(1, 1000:1500))) '\t\t''\t\t''\n'])

%cloneDivSlope = LinearModel.fit(2000:100:4000, cloneDivAver6(1, 2000:100:4000))

%\\\\\\\\\\\\\\\\\\\\\\\\\\\\\\\\\\\\\\\\\\\\\\\\\\\\\\\\\\\\

%/////////////////////////////////////////////////////////////

% AVERAGING MUTATION ACCUMULATION SLOPE FROM 6 PATIENTS

mutAver6 = zeros(1, size(totMut, 2));

for i = 1 : size(totMut, 2)

mutAver6(1, i) = mean(totMut(:, i));

end

mdl = LinearModel.fit(2000:4000, mutAver6(1, 2000:4000))

%disp('sigma: mutation fold increase:')

%fprintf([num2str(sigma) '\t\t''\t\t' num2str(mutRateFoldIncrease) '\n'])

%\\\\\\\\\\\\\\\\\\\\\\\\\\\\\\\\\\\\\\\\\\\\\\\\\\\\\\\\\\\\\\\\

%TRACKING CLONAL EXPANSIONS

maxClonAver6 = zeros(1, size(maxClon, 2));

for i = 1 : size(maxClon, 2)

maxClonAver6(i) = mean(maxClon(:, i));

end

%disp(std(maxClon(:, end)))

%disp(mean(maxClon(:, end)))

%plot(1 : maxAge, maxClonAver6)

maxClon19 = [];

k = 0;

for i = 1 : 232 : size(maxClonAver6, 2)

k = k+1;

if i < 4177

maxClon19(k) = mean(maxClonAver6(1, i:i+231));

else

maxClon19(k) = mean(maxClonAver6(1, i:end));

break;

end

end

disp('max terminal expansion % POOL:')

disp(maxClon19(1, end))

%plot(1 : size(maxClon19, 2), maxClon19)

for i = 1 : size(maxClon19, 2)

maxClon19(1, i) = (maxClon19(1, i) - min(maxClon19))/(max(maxClon19) - min(maxClon19));

end

maxClon19 = maxClon19.';

similarityRMSE = sqrt(sum((maxClon19(:, 1) - leukem(:, 1)).^2)/size(leukem, 1));

disp('curve similarity:')

fprintf([num2str(1 - similarityRMSE) ' %' '\n\n'])

disp('');

plot(1:19, maxClon19, 1:19, leukem)

disp('sigma: mutation fold increase:')

fprintf([num2str(sigma) '\t\t''\t\t' num2str(mutRateFoldIncrease) '\n'])

%///////////////////////////////////////////////////////////////////////////////////

%

%average fitness drop

%plot(1:4420, averFitn)

%disp('minimum fitness across the board')

%disp(min(averFitn))

%disp('')

%disp('')

%disp('minimum terminal fitness')

%disp(averFitn(end))

**Code for initial matrix transformation by interpolating intermediate values (spline matrices):**

matrix = A_pastespecial;

mutR = [1 : size(matrix, 2)];

%σ’s = [1, 2, 3, 4, 5, 6, 7, 8, 9];

SDs = [1 : size(matrix, 1)];

SDs = fliplr(SDs);

splinMatrixSizeMut = [1 : 0.01 : size(matrix, 2)];

splinCADsimilar1 = zeros(size(matrix, 1), size(splinMatrixSizeMut, 2));

for i = 1 : size(matrix, 1)

mutRi = mutR.';

CAD = matrix(i, :).';

splin = fit(mutRi, CAD, 'pchipinterp');

splinR = feval(splin, splinMatrixSizeMut);

splinR = splinR.';

splinCADsimilar1(i, :) = splinR;

end

splinMatrixSizeSD = [1 : 0.01 : size(matrix, 1)];

splinCADsimilar2 = zeros(size(splinMatrixSizeSD, 2), size(splinMatrixSizeMut, 2));

for i = 1 : size(splinCADsimilar1, 2)

SDsi = SDs.';

CAD = splinCADsimilar1(:, i);

splinSD = fit(SDsi, CAD, 'pchipinterp');

splinRSD = feval(splinSD, splinMatrixSizeSD);

splinCADsimilar2(:, i) = splinRSD;

end

levels = [0.03759 0.1457];

cont = contourf(splinMatrixSizeMut, splinMatrixSizeSD, splinCADsimilar2, 20)

hold on

%contour(splinMatrixSizeMut, splinMatrixSizeSD, splinCADsimilar2, levels)

%clabel(cont, levels)

%clabel(cont)

hold off

%CADtenthexp = splinCADsimilar2;

clearvars A_pastespecial matrix mutR SDs splinMatrixSizeMut splinCADsimilar1...

i mutRi CAD splin splinR splinMatrixSizeSD SDsi splinSD...

splinRSD levels cont

%splinCADsimilar2

**Code for calculating surface stats (Presented in Table 1 of the main text):**

matrixPlaus = ENV0plaus; %a spline-transformed matrix of mutation accumulation slopes

matrixSimil = ENV0simil; % a spline-transformed matrix of curve similarity

matrixExp = ENV0exp; % a spline-transformed matrix of the magnitude of terminal clonal expansions

levels = [0.03759 0.1457]; % confidence intervals for the reference WGS Tier 3 mutation accumulation slope

plausArea = 0;

for i = 1 : size(matrixPlaus, 2)

plausArea = plausArea + numel(matrixPlaus(matrixPlaus(:, i) >= levels(1) & matrixPlaus(:, i) <= levels(2), i));

end

similArea = 0;

cutoff = 0.7;

for i = 1 : size(matrixSimil, 2)

similArea = similArea + numel(matrixSimil(matrixSimil(:, i) >= cutoff));

end

similAreaOverlap = 0;

for i = 1 : size(matrixSimil, 1)

for k = 1 : size(matrixSimil, 2)

if matrixSimil(i, k) >= cutoff & matrixPlaus(i, k) >= levels(1) & matrixPlaus(i, k) <= levels(2)

similAreaOverlap = similAreaOverlap + 1;

end

end

end

expVolume = 0;

for i = 1 : size(matrixExp, 1)

for k = 1 : size(matrixExp, 2)

if matrixPlaus(i, k) >= levels(1) & matrixPlaus(i, k) <= levels(2)...

& matrixSimil(i, k) >= cutoff

expVolume = expVolume + matrixExp(i, k);

end

end

end

disp('plausible area, %:')

disp(plausArea/numel(matrixPlaus)*100)

disp('above cutoff similarity area, %:')

disp(similArea/numel(matrixSimil)*100)

disp('area of overlap, %:')

disp(similAreaOverlap/(plausArea+similArea-similAreaOverlap)*100)

disp('volume of expansions above overlap:')

disp(expVolume)

disp('average expansion % pool within overlap:')

disp(expVolume/similAreaOverlap)
